# Microglia Single‐Cell RNA‐Seq Enables Robust and Applicable Markers of Biological Aging

**DOI:** 10.1111/acel.70095

**Published:** 2025-05-15

**Authors:** Natalie Stanley, Luvna Dhawka, Sneha Jaikumar, Yu‐Chen Huang, Anthony S. Zannas

**Affiliations:** ^1^ Department of Computer Science and Computational Medicine Program The University of North Carolina at Chapel Hill Chapel Hill North Carolina USA; ^2^ Department of Genetics The University of North Carolina at Chapel Hill Chapel Hill North Carolina USA; ^3^ Curriculum in Bioinformatics and Computational Biology The University of North Carolina at Chapel Hill Chapel Hill North Carolina USA; ^4^ Department of Psychiatry The University of North Carolina at Chapel Hill Chapel Hill North Carolina USA

**Keywords:** markers of biological aging, microglia, single‐cell RNA sequencing, transcriptomics

## Abstract

“Biological aging clocks”—composite molecular markers thought to capture an individual's biological age—have been traditionally developed through bulk‐level analyses of mixed cells and tissues. However, recent evidence highlights the importance of gaining single‐cell‐level insights into the aging process. Microglia are key immune cells in the brain shown to adapt functionally in aging and disease. Recent studies have generated single‐cell RNA‐sequencing (scRNA‐seq) datasets that transcriptionally profile microglia during aging and development. Leveraging such datasets in humans and mice, we develop and compare computational approaches for generating transcriptome‐wide summaries from microglia to establish robust and applicable aging clocks. Our results reveal that unsupervised, frequency‐based summarization approaches, which encode distributions of cells across molecular subtypes, strike a balance in accuracy, interpretability, and computational efficiency. Notably, our computationally derived microglia markers achieve strong accuracy in predicting chronological age across three diverse single‐cell datasets, suggesting that microglia exhibit characteristic changes in gene expression during aging and development that can be computationally summarized to create robust markers of biological aging. We further extrapolate and demonstrate the applicability of single‐cell‐based microglia clocks to readily available bulk RNA‐seq data with an environmental input (early life stress), indicating the potential for broad utility of our models across genomic modalities and for testing hypotheses about how environmental inputs affect brain age. Such single‐cell‐derived markers can yield insights into the determinants of brain aging, ultimately promoting interventions that beneficially modulate health and disease trajectories.

## Graphical abstract

This study develops computational approaches to translate single‐cell based transcriptomic signatures of microglia into aging clocks, or machine learning models of age. These models can be broadly applied to new single‐cell or bulk RNA‐sequencing datasets to gain novel biological insights and evaluate how environmental inputs (such as stress) affect brain aging.

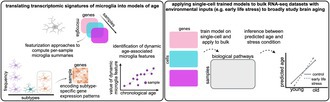

## 1 Introduction

An exciting advancement over the recent years has been the development of “biological aging clocks,” composite molecular markers that are thought to capture the rate at which an individual ages biologically (Horvath and Raj 2018; Zannas 2019). Initially developed as predictors of chronological age (Horvath 2013; Hannum et al. 2013), the subsequently developed clocks were further shown to predict diverse aging‐related disease and mortality outcomes (Lu et al. 2019; Levine et al. 2018; Belsky et al. 2022), supporting their promise as disease biomarkers. To date, aging clocks have been generated by various molecular platforms—for example, transcriptomics, epigenomics, proteomics—largely applied in data combined from many tissues and organs or in heterogeneous mixtures of cells from single tissues (i.e., at the bulk level) (Horvath 2013; Vijayakumar and Cho 2022). While such bulk‐level analyses are essential for biomarker discovery, molecular changes and aging rates are known to vary across different tissues and cell types (Horvath et al. 2015; Sonawane et al. 2017; Kabacik et al. 2022). Moreover, disease‐related alterations in aging markers and their potential underlying pathways have been reported to occur in a tissue‐ and cell type‐specific manner (Horvath et al. 2014; Coninx et al. 2020; Dulken et al. 2019), highlighting the importance of gaining cell‐level insights into biological aging.

Microglia are key immune cells in the brain (Aloisi 2001; Colonna and Butovsky 2017) and have been critically implicated in the neuroinflammation associated with aging (Sierra et al. 2007; Mosher and Wyss‐Coray 2014), adaptations to stress (Koo and Wohleb 2021; Biltz et al. 2022), neurodegenerative states (Prater et al. 2023; Song and Colonna 2018; Chen et al. 2023), and diverse neuropsychiatric disorders (Singhal and Baune 2017; Prinz and Priller 2014). In these processes, microglia can undergo dynamic transcriptional alterations that are relevant to brain function and disease pathogenesis (Koo and Wohleb 2021; Chen et al. 2023; Bolte and Lukens 2021). In particular, microglia have been shown to exhibit several hallmarks of aging, including shortened telomeres, loss of proteostasis, aberrant intercellular communication, and altered cellular morphology and phagocytosis (Fan et al. 2024; Shea and Villeda 2024). Recent seminal single‐cell RNA‐sequencing (scRNA‐seq) studies further suggest that such alterations involve specific microglia subtypes and key genetic programs that can be leveraged by machine learning algorithms to develop microglia aging markers (Hammond et al. 2019; Buckley et al. 2023; Keren‐Shaul et al. 2017). Importantly, functional alterations in specific microglia subtypes are also associated with distinct aging‐related brain phenotypes, including neural stem cell proliferation (Buckley et al. 2023), neurodegeneration (Silvin et al. 2022), and Alzheimer's disease (Keren‐Shaul et al. 2017). Therefore, uncovering the transcriptional dynamics of microglia at the single‐cell and cell‐type levels may yield unique insights into the determinants of brain aging and disease.

With single‐cell technologies offering an unprecedented level of resolution in characterizing functional states of individual cells, quality computational methods are required to translate such information into machine learning models of sample‐level phenotype. Here, featurization refers to the process of translating multiple prominent gene expression patterns and relative cell‐type abundances from single‐cell datasets into succinct summaries that can be input to machine learning models of aging. Practically, each sample profiled with a single‐cell technology produces a large matrix of many features measured per individual cell, which must be translated into a sample‐level feature vector to ultimately train machine learning models of age.

Computational featurizations of single‐cell data have been explored substantially for translating abundances and functional states of immune cell types assayed with flow and mass cytometry (Stanley et al. 2020; Bruggner et al. 2014; Hu et al. 2019; Shan et al. 2022; Yi and Stanley 2021, 2023; Arvaniti and Claassen 2017; Chen et al. 2024), but these approaches cannot always be directly applied to analyze high‐dimensional scRNA‐seq data. scRNA‐seq based transcriptomic clocks derived from pseudobulk features (Buckley et al. 2023; Zakar‐Polyák et al. 2024), transcriptome binarization (Meyer and Schumacher 2021), or cell‐type compositions (Zhu et al. 2023; Mao et al. 2023) have been successfully applied to identify critical aging‐linked immunological changes in humans, mice, and *C. elegans* . While supervised featurization methods can be trained to learn per‐sample representations, based on external information such as age (Yi and Stanley 2021; Arvaniti and Claassen 2017), here we specifically examine the capacity of unsupervised feature engineering strategies, such as computing cell‐type frequencies (Stanley et al. 2020) or pseudobulk‐level features (Junttila et al. 2022), to be used to train single‐cell microglia‐based models of age. Such unsupervised approaches are well suited for datasets with small sample sizes and for uncovering meaningful patterns that are biologically interpretable, such that they can illuminate cell types and their gene regulatory programs associated with particular aging trajectories. While Buckley et al. (2023) pioneered the use of a pseudobulk‐based approach to compute per‐sample featurizations across a range of cell types in the brain, it has not been adequately explored how the range of possible featurization approaches can translate cellular heterogeneity patterns into models of age. Because age‐related biological processes occur in a tissue‐ and cell type‐specific manner (Horvath et al. 2014; Coninx et al. 2020; Dulken et al. 2019), we hypothesize that implementing featurization in specific signal‐rich cell types, such as microglia, can increase the biological signal‐to‐noise ratio, leading to aging markers with enhanced robustness and applicability.

**FIGURE 1:**
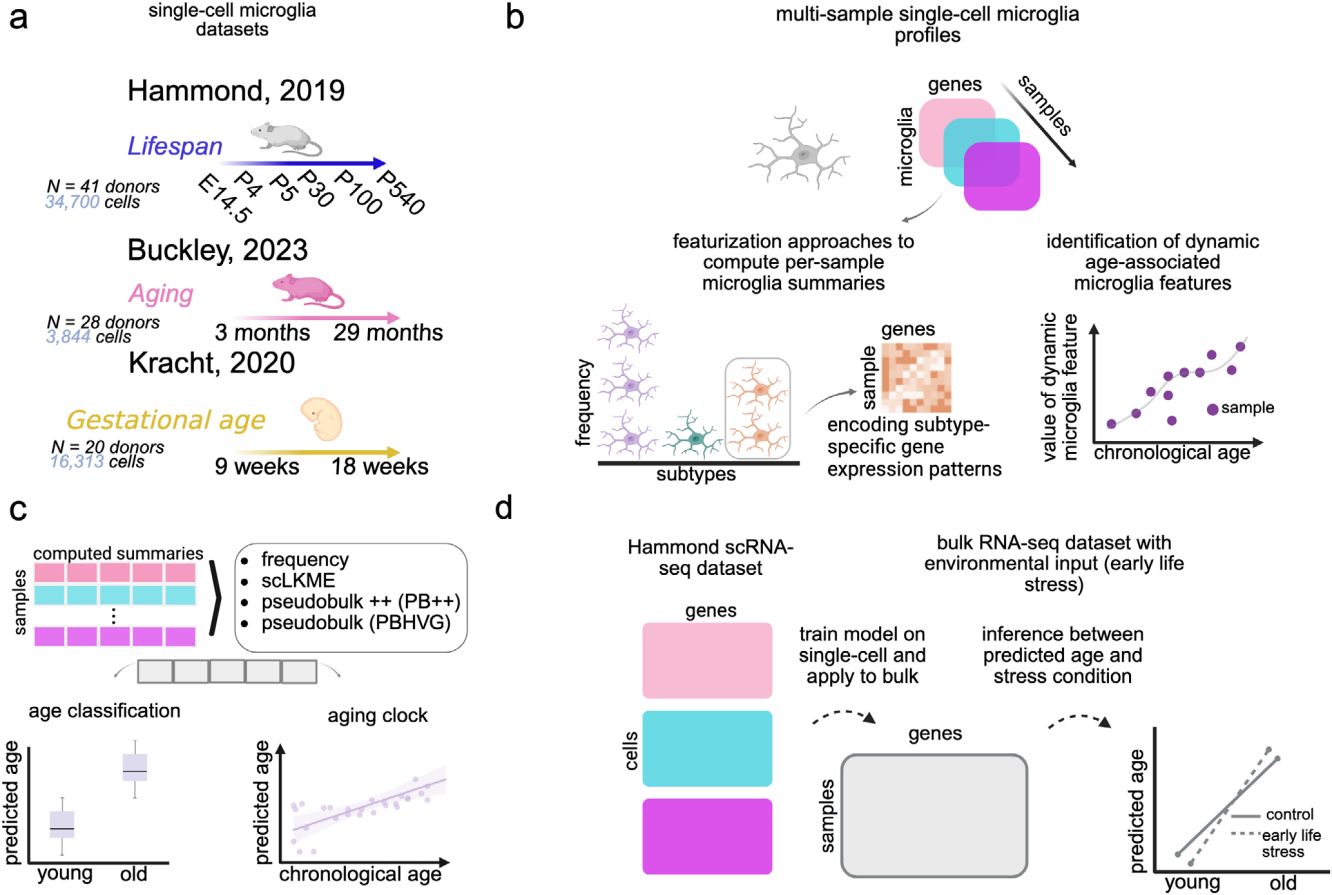
Overview. (a) We used three scRNA‐seq datasets profiling microglia during aging and development to build microglia aging clocks. The Hammond dataset has six discrete time points spanning between embryonic stage (E14.5) and old age (P540). The Buckley and Kracht datasets have continuously sampled ages between 3 and 29 months and 9 and 18 gestational weeks, respectively. The schematic denotes the number of donors (*N*) and the number of cells profiled in each study. (b) To ultimately build aging clocks, or machine learning models of age, we computed computational summaries or *featurizations* for each sample based on frequencies and genetic programs of microglia subtypes. (c) Four approaches for featurizing or summarizing transcriptomic patterns of microglia in profiled samples were applied and include frequency, single‐cell landmark kernel mean embedding (scLKME), pseudobulk++ (PB++), and classical pseudobulk (PBHVG). The various featurization methods generate succinct features that can be used as input to machine learning models of age. (d) A model for age was trained on the Hammond single‐cell data and applied to an independent bulk RNA‐seq dataset with an additional environmental input (exposure to early life stress).

While aging and development involve distinct biological processes, they also represent a continuum of the passage of “biological time,” and including datasets that fully capture this continuum can provide important insights into the computational principles required to model biological aging. Therefore, in this study we leveraged publicly available scRNA‐seq datasets profiling multiple microglia samples across the lifespan (Figure 1a) to pursue three main objectives. First, we determined the extent to which frequencies of particular microglia subtypes and their gene expression patterns change dynamically during aging and development (Figure 1b). Using the same datasets, we also quantified how four methodologically diverse, unsupervised featurization approaches of microglia transcriptome‐wide signatures perform as age classifiers and aging clocks (Figure 1c). Lastly, we assessed whether the newly constructed microglia clocks and their conserved genetic programs are applicable to bulk RNA‐seq data with environmental inputs (Figure 1d). In doing so, we show that the single‐cell based signatures can extend well to bulk RNA‐seq datasets for broad applicability in future aging studies. Overall, our findings indicate that single cell‐level and cell type‐specific computational approaches yield robust microglia markers of aging that are applicable across diverse datasets and provide unique, biologically relevant insights into brain aging.

## 2 Results

To understand the tradeoffs in accuracy, interpretability, and efficiency of different featurization approaches, we leveraged three multisample scRNA‐seq datasets that profile microglia transcriptionally during lifespan (Hammond et al. 2019), aging (Buckley et al. 2023), and fetal development (Kracht et al. 2020) (Figure 1a). Key microglia subtypes and their genetic programs identified under the different featurization approaches were leveraged to generate transcriptomic signatures of microglia in aging and to compare such signatures across multiple datasets. To assess whether the findings uncovered through single‐cell analysis can be generalized at the bulk level, the prominent genes identified by scRNA‐seq were validated and used to train an aging clock that could be broadly applied to an additional bulk RNA‐seq dataset of young (P9) and old (P200) mice generated by Reemst et al. (2022).

### 2.1 Single‐Cell RNA‐Sequencing Datasets of Microglia During Aging and Development

To enable a comprehensive comparison of single‐cell based featurization approaches (Figure 1b) for generating robust and accurate aging clocks from transcriptomic signatures of microglia, we chose three diverse datasets (Figure 1a) from mice, humans, and different brain regions in the contexts of both aging and development: (1) The Hammond mouse lifespan dataset profiled mice throughout the lifespan (Hammond et al. 2019), with samples collected from whole brain at embryonic Day 14.5 (E14.5) and postnatal Days 4 (P4), 5 (P5), 30 (P30), 100 (P100), and 540 (P540); (2) The Buckley mouse aging dataset (Buckley et al. 2023) sampled microglia from the subventricular zone (SVZ) neurogenic region at different ages between 3.3 and 29 months; (3) The Kracht fetal development dataset (Kracht et al. 2020) profiled microglia that were isolated from CNS tissue from aborted human fetuses, obtained at timepoints sampled at different ages between 9 and 18 weeks of gestational age. More detailed descriptions of these datasets and preprocessing techniques can be found in Section 4.

### 2.2 Microglia Subtype Frequencies Vary With Age

**FIGURE 2:**
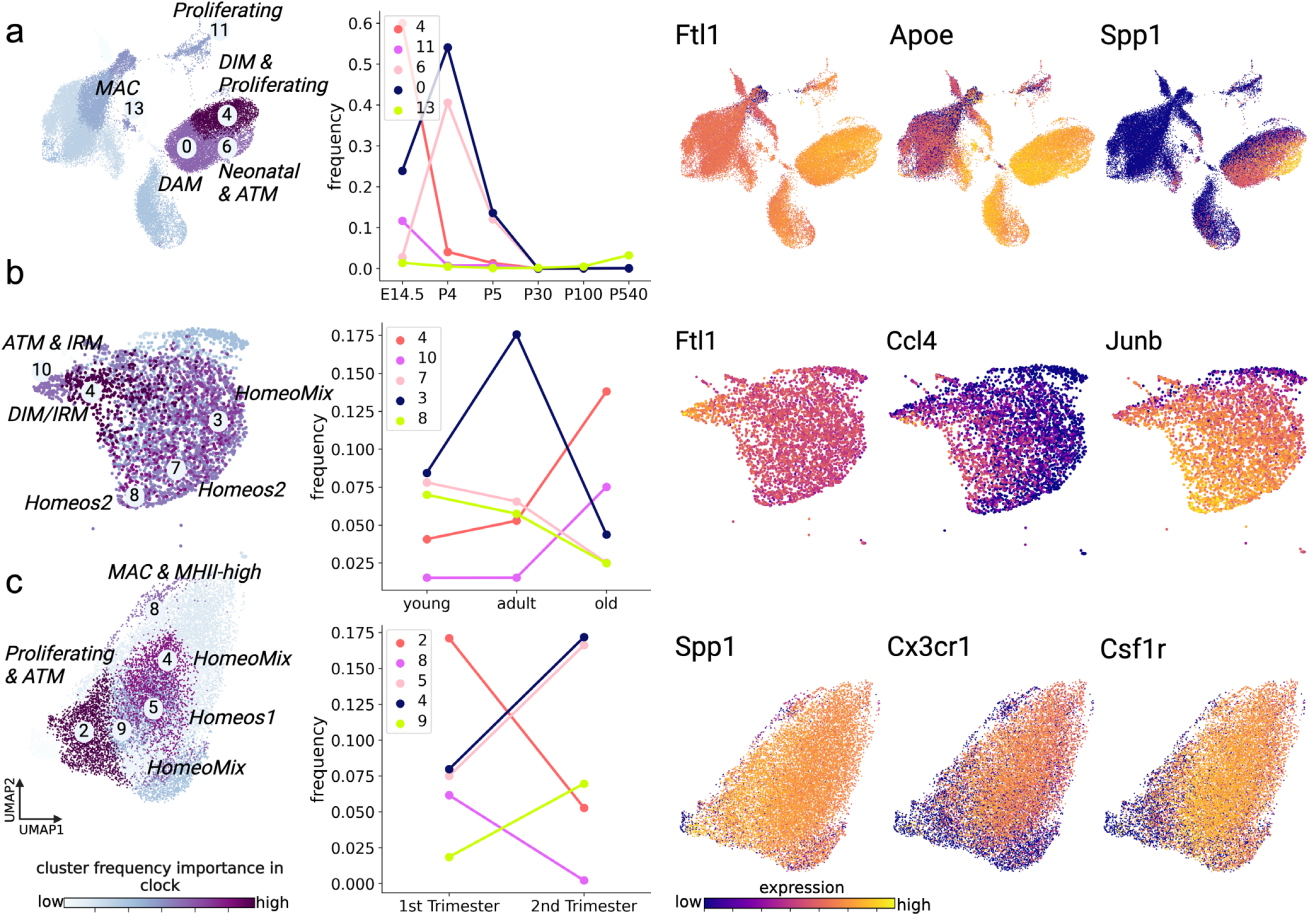
Frequencies of microglia subtypes change dynamically during aging and development. Age‐related changes in microglia subtype frequencies were observed across the Hammond (a), Buckley (b), and Kracht (c) datasets. Left panels show two‐dimensional UMAP projections of cells in each dataset. Cells are colored by the usefulness of their respective cluster's frequency in predicting age (light to dark indicates less to more useful), according to cluster frequency Gini score computed for a random forest classifier trained to predict discrete age groups. Discrete age groups were defined for all datasets (further details in Section 4). Five representative clusters with critical dynamic changes in frequency useful for age‐classification are depicted and numbered in each UMAP plot. Clusters were manually annotated into subtypes according to marker genes described in previous studies (defined in Tables S1–S3). Clusters labeled as HomeoMix are mixtures of Homeostatic 1, Homeostatic 2, and Homeostatic 3 (see descriptions of homeostatic subtypes in Ref. (Martins‐Ferreira et al. 2025)). Middle panels plot the trajectory of mean frequencies of the top five age‐predictive clusters in each dataset as a function of age group. Right panels show two‐dimensional UMAP projections of cells in each dataset colored by the expression (dark to light indicates low to high expression) of key genes that were differentially expressed in at least one of the five prioritized clusters.

All three datasets exhibited microglia heterogeneity based on Leiden clustering (Traag et al. 2019) that identified diverse microglia subtypes with characteristic gene expression programs (Figure S1). As a first approach to link cell heterogeneity to age, we examined how the frequencies of identified microglia subtypes varied over the aging trajectory in each dataset. Cluster‐to‐subtype annotations were performed manually based on previous studies (Hammond et al. 2019; Keren‐Shaul et al. 2017; Silvin et al. 2022; Martins‐Ferreira et al. 2025; Masuda et al. 2019; Galatro et al. 2017; Gerrits et al. 2020; Marschallinger et al. 2020; Frigerio et al. 2019; Mathys et al. 2017; Mrdjen et al. 2018; Wlodarczyk et al. 2017) and are provided in Tables S1–S3. Subtypes from the literature are described in the Supporting Information Table entitled Microglia_Subtype_Stanley_Dhawka_Zannas.csv, and per‐cluster expression patterns in each dataset are visualized in Figure S17. To link microglia heterogeneity to age, we computed frequency features as the fraction of each sample's cells assigned to each cluster according to previously described methods (Stanley et al. 2020). To identify the microglia subtypes that most strongly associate with age, subtype frequencies were then used to train and test a Random Forest classifier for age group across 200 trials (see Section 4), and mean Gini (Menze et al. 2009) scores were computed as a metric of frequency feature importance (Figure 2). Figure 2 left visualizes cells from the Hammond, Buckley, and Kracht datasets (a–c, respectively). Cells are colored by the inferred importance of the frequency of the cluster to which they belong. In each dataset, we further highlighted the trajectories of the top five clusters with predictive and dynamic frequencies across the age spectrum (Figure 2 middle). In the Hammond dataset, Clusters 4 and 11 exhibited similar patterns, having high frequencies at embryonic stage (E14.5) and then attenuating at older ages. Cluster 4 showed high expression of *Ftl1*, *Apoe*, and *Ctsb* (Figure 2a, right, Figure S8), whereas cluster 11 was marked prominently by *Stmn1*, *Tubb5*, and *Tuba1a*. Cluster 13 increased in frequency with age and was marked by *Malat1*, *Apoe*, and *Ifitm3*. In the Buckley dataset, Clusters 4 and 10 exhibited very similar gene expression patterns with frequencies that significantly increased between adult and old age. These were marked by expression of *Fth1*, *Ftl1*, and *Ctsb* (Figure 2b, right, Figure S9). Clusters 7 and 8 also had distinct gene expression patterns with frequencies that were highest at young and adult ages but decreased in old age. These clusters were prominently marked by transcription factors *Jun* and *Junb*, as well as by *Malat1*. Finally, in the Kracht dataset, Clusters 5, 4, and 9 increased in frequency between the first and second trimesters and were marked by expression of *Cx3cr1*, *Ftl*, and *Csf1r* (Figure 2c, right, Figure S10), whereas Clusters 2 and 8 decreased in frequency with age and both expressed *Malat1*, *Ftl*, and *Spp1*. Together these analyses indicate dynamic changes in microglia subtypes and gene expression signatures across aging and developmental stages (a visualization of the trajectories of subtype frequencies across datasets is shown in Figure S11).

### 2.3 Featurization Methods Impact the Accuracy of Microglia Aging Markers

With evidence that microglia subtypes change over the aging continuum, we next explored a range of strategies to generate aging markers derived from scRNA‐seq data. While clocks have historically been developed by modeling age as a continuous variable, we evaluated the usefulness of each featurization approach in both classifying age groups (i.e., discrete age variable) and predicting age (i.e., continuous age variable), through classification and regression models, respectively. More specifically, we examined how four methodologically distinct featurization techniques can be applied to translate a multisample single‐cell dataset into a succinct vector representation or transcriptome‐wide summary that can be used as input to a machine learning model of age (Figure 1c). We provide a description of each featurization algorithm here and provide further technical details in the Section 4.

#### 2.3.1 Frequency

Frequency‐based featurization (Stanley et al. 2020; Bruggner et al. 2014) involves defining a common set of microglia subtypes through unsupervised clustering of all cells across samples within a dataset. A feature representation is computed for each sample by counting the fraction of its cells assigned to each subtype.

#### 2.3.2 scLKME

scLKME (Yi and Stanley 2023) is an unsupervised feature learning approach, which encodes the complexity of the single‐cell landscape into a kernel‐based feature representation that can be used as input to classification models. scLKME performs landmark‐based kernel mean embedding, where a set of landmarks or generally prototypical cells are chosen across all samples. Sample feature representations are ultimately encoded based on similarity patterns of each sample's cells to the set of landmark cells, as evaluated through a kernel evaluation (Shan et al. 2022).

#### 2.3.3 Pseudobulk Based on Highly Variable Genes (PBHVG)

Pseudobulk representations of single‐cell data are the most common approach for summarizing broad gene expression patterns in single‐cell data to estimate bulk signatures. While there are a range of techniques for creating pseudobulk profiles, outlined comprehensively in Ref. (Junttila et al. 2022), here we computed the sum of each gene's expression in each cluster. Because considering the expression of all measured genes would create a feature space with a prohibitively high dimensionality, we considered only the expression of the 3,000 most highly variable genes (HVG) in each cluster, which is a common preprocessing step in many single‐cell pipelines (Korsunsky et al. 2019). This also implies that there are 3,000 generated pseudobulk features per‐cell cluster.

#### 2.3.4 Pseudobulk++ (PB++)

In contrast to a traditional pseudobulk approach (Junttila et al. 2022), which computes aggregate expression measurements for all genes across all cells in each cluster, the pseudobulk (PB)++ approach computes a single value for each cluster, which reflects the general frequency and alignment with key gene expression programs of cells in the sample. Specifically, PB++ variants integrate frequency information (i.e., the number of cells across major cell‐populations) with the extent to which key genes are expressed in each cluster. In the PB++25 and PB++50 variants, we use the top 25 and 50 differentially expressed genes across clusters, respectively. This newly proposed method aims to parsimoniously integrate signals originating from cell type frequencies and key gene expression programs.

### 2.4 Microglia Age Classifiers

**FIGURE 3:**
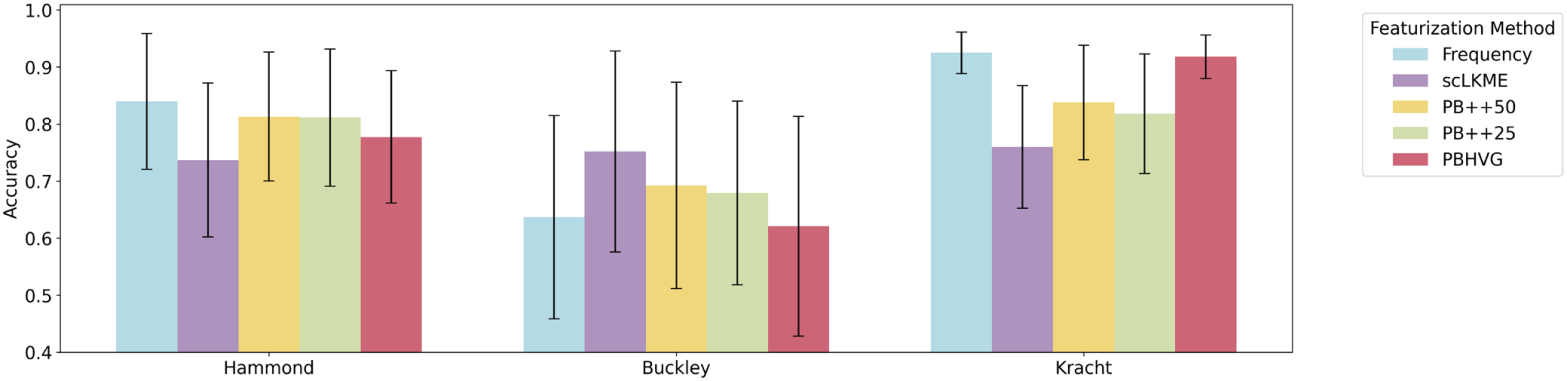
Accuracy of microglia aging classifiers on predicting the age of each donor across featurization approaches. Discrete age groups were created in each dataset to formulate a discrete age classification problem, using the various featurizations, including frequency, scLKME, Pseudobulk++50 (PB++50), Pseudobulk++25 (PB++25), and pseudobulk with highly variable genes (PBHVG) as input. Classification experiments were performed using random forest classifiers and repeated 200 times with randomized train/test splits of donors. Barplots and error bars show the mean and standard deviation in classification accuracy obtained over the 200 trials. Details on discrete age categories specified for each dataset are provided in Section 4.

To assess classification performance across featurization approaches, ages in each dataset were binned into discrete categories, based on age group (Hammond), age distribution (Buckley), or trimester of pregnancy (Kracht). Histograms of age distributions are shown in Figure S6. Note that the Hammond dataset already had discrete age categories (embryonic stage E14.5, P4, P5, P30, P100, and P540). The Buckley dataset was discretized into young (< 4 months), adult (4 months to less than 14 months), and old (≥ 14 months and above). The Kracht dataset was binned by trimester, such that the first and second trimesters contained samples of ages 9–12 and 13–18 weeks, respectively. Classification experiments involved 200 trials of random train/test splits, where 80% of donors and their respective samples were used for training and the remaining 20% for testing model accuracy (Acc), defined as the fraction of donors with a correctly predicted age group. Figure 3 shows the results of the classification experiments across featurization approaches and datasets. In the Hammond dataset, which has the largest number of donors and age groups, the top performing methods were frequency and PB++, resulting in mean accuracies of Acc = 0.840 and Acc = 0.813, respectively. This suggests that larger datasets are sufficiently well‐powered to use these simple, low‐dimensional featurization approaches. In the Buckley dataset, which has the smallest number of cells overall, the top performing method was scLKME (mean Acc = 0.752), followed by PB++50 (mean Acc = 0.693). This suggests that features based on similarity patterns with landmark or anchor cells contained enough signal to accommodate smaller datasets. The Kracht dataset achieved strong performance with frequency features (mean Acc = 0.925), followed by PBHVG (mean Acc = 0.918). In each of the three datasets, either frequency or a PB++ variant achieved the top two highest classification accuracies. Such methods produce feature representations that are the lowest dimensional, with the number of features corresponding to the number of clusters or inferred microglia subtypes. Therefore, our results suggest robust classification performance by simple and computationally efficient approaches, such as frequency or PB++ variants across aging and development. Figure S15 shows Jaccard similarity between the top 10 most predictive features across pairs of frequency‐based featurizations (including frequency, PB++25, and PB++50) and reveals that the three methods leverage similar subtype frequencies as key signals in their respective models. However, there are differences between top predictive features under the frequency and PB++ variants, likely due to having included gene expression signatures in defining features.

### 2.5 Microglia Aging Clocks

**FIGURE 4:**
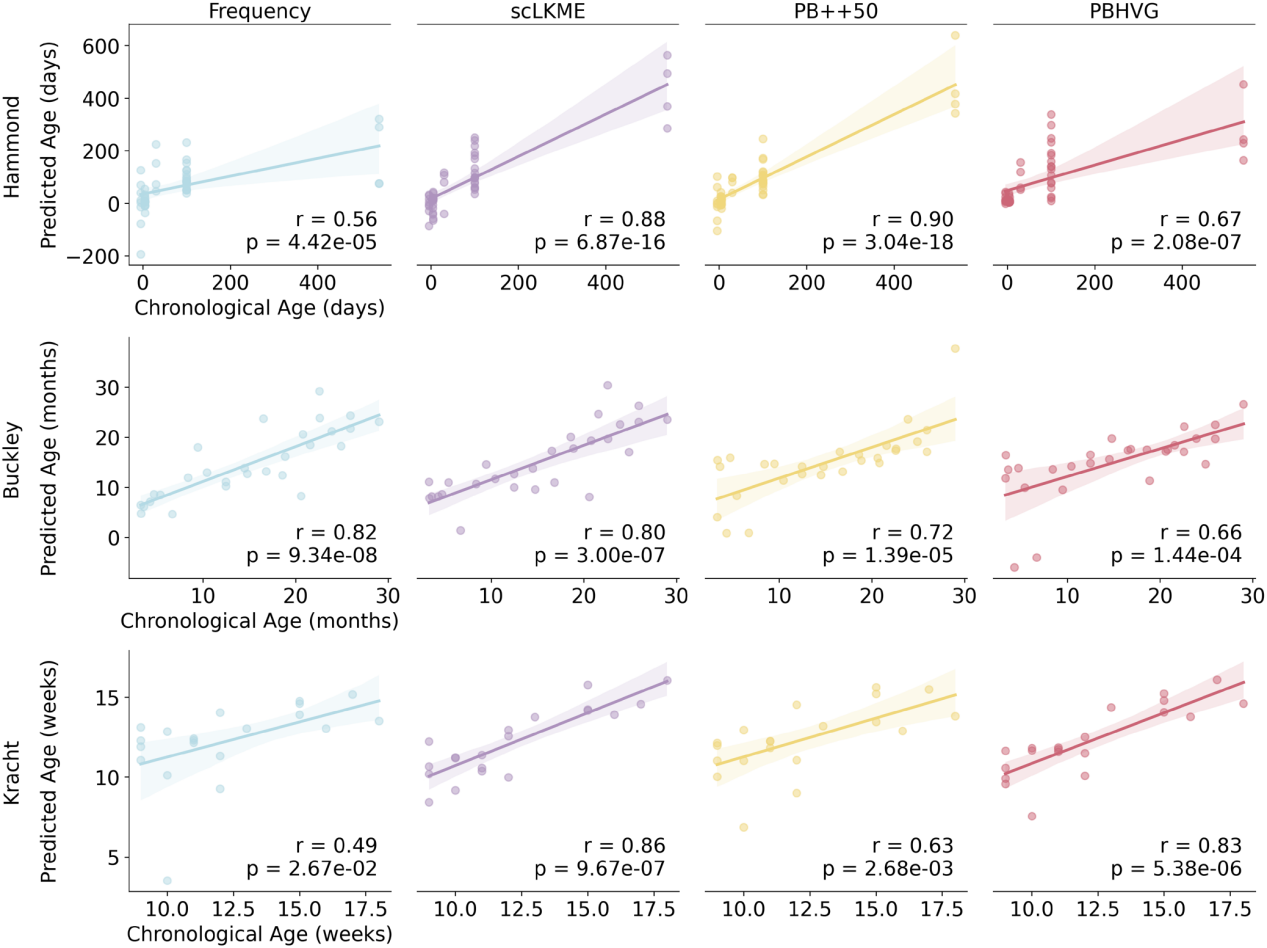
Performance of microglia aging clocks on predicting donor ages across featurization approaches. Age was used as a continuous variable to train Lasso regression models under frequency, scLKME, Pseudobulk++50 (PB++50), and pseudobulk with highly variable genes (PBHVG) featurization approaches, using leave‐one‐out cross‐validation (LOOCV) in the Hammond, Buckley, and Kracht datasets. Scatterplots show the predicted versus true chronological age for each sample obtained in the LOOCV trial for which it was the test sample. Note that the Kracht dataset has multiple sample batches per donor, so each point depicts their mean predicted age for each donor. Correlation coefficients between chronological and predicted ages are quantified with Pearson correlation (*r*).

To generate microglia aging clocks (i.e., predictors of chronological age), we employed a leave‐one‐out cross‐validation approach that used all but one donor and their respective samples to train a Lasso Regression model on a given set of features. We then evaluated the predicted age of the held‐out donor and plotted them against their chronological age in Figure 4. In the Hammond dataset (Figure 4 top), PB++50 showed the highest correlation between chronological and predicted ages (*r* = 0.90, *p* = 3.04 × 10^−18^), followed by scLKME (*r* = 0.88, *p* < 6.87 × 10^−16^). In the Buckley dataset (Figure 4 middle), frequency showed the highest concordance between chronological and predicted age (*r* = 0.82, *p* = 9.3 × 10^−8^), with scLKME as the second top‐performing method (*r* = 0.80, *p* = 3 × 10^−7^), suggesting that the additional information content included through a higher‐dimensional feature space was only marginally helpful. In the Kracht dataset, the regression approach was more challenging, given the limited age range (Figure 4 bottom). scLKME achieved the highest accuracy (*r* = 0.86, *p* = 9.67 × 10^−7^), followed by PBHVG (*r* = 0.83, *p* = 5.38 × 10^−6^).

Overall, the results suggest that for datasets covering a more extended age range (like Hammond and Buckley), scLKME is the most robust choice, but lower‐dimensional methods such as PB++50 and frequency can also achieve robust prediction accuracies. In datasets with a more limited age range (like Kracht), higher‐dimensional approaches such as PBHVG and scLKME may be necessary to maximize the signal‐to‐noise ratio necessary for robust prediction. It is also worth noting that frequency features showed the weakest performance in the Hammond and Kracht datasets, despite excelling in age classification (Figure 3), suggesting these features may lack adequate transcriptional signal for the regression task. This is especially true in the Kracht dataset, where we hypothesize that the limited age range is not discernible through frequency features alone. PB++ features were advantageous in the Kracht and Hammond datasets in decreasing prediction variance. This suggests that the addition of gene expression information adds useful signal when the training data have compressed age ranges (as in Kracht) or are not uniformly sampled across the lifespan (as in Hammond).

### 2.6 Computational Requirements of Featurization Approaches

We next compared the computational requirements in terms of both run‐time and memory across the featurization approaches (with run‐time and memory requirements shown in Figure S7). The variation in run‐time and in the resources required for age classification and prediction was primarily driven by the number of features produced under each approach (Table 1). Figure S7 shows mean run‐time (panels a and c) and memory requirements (panels b and d) for clocks constructed based on classification (panels a and b) and regression (panels c and d). Results were obtained on a machine with Intel(R) Xeon(R) Gold 6226R CPU @ 2.90GHz. Frequency was unequivocally the most efficient method, both in terms of run‐time and memory requirements, since it is an inherently low‐dimensional featurization technique that produces as many features as the number of clusters. The PB++ variants take slightly longer to compute during the featurization step than PBHVG but lead to an overall reduced run‐time and memory requirements for the overall pipeline as they produce a low‐dimensional space (number of features also equal to the number of clusters). PBHVG produces a large number of features, increasing the run‐time and memory requirements. Finally, scLKME has the highest run‐time and memory requirements across datasets, likely due to the kernel evaluations built into the method and the high‐dimensional feature space produced.

**TABLE 1:** The number of features generated under each combination of dataset and featurization approach.

	Hammond	Buckley	Kracht
Frequency	17	14	13
scLKME	1500	1500	1500
PB++50	17	14	13
Pseudobulk (HVG)	51,000	42,000	21,905

### 2.7 Cross‐Dataset Integration and Independent Single‐Cell Validation of Models

To evaluate the potential for broad use of the aging clocks in future aging studies, we performed cross‐dataset training–testing procedures in mice. In such experiments, models were trained on one dataset and tested in an independent dataset. For evaluation, we also leveraged *N* = 6 samples from wildtype aging mice from the Keren‐Shaul study (Keren‐Shaul et al. 2017) at 49 and 600 days of life.

**FIGURE 5:**
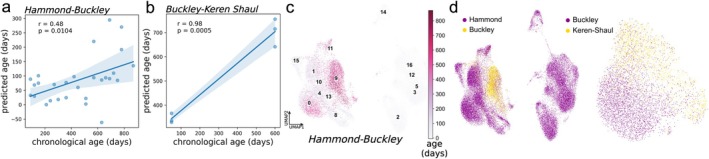
Cross‐dataset integration and evaluation. Microglia‐based aging clocks trained on individual studies were evaluated on microglia in different studies. (a) Microglia subtypes from the Hammond study were mapped to cells in the Buckley dataset and used to define frequency features to train and test a model in the Hammond and Buckley studies, respectively. Plots show the chronological versus the predicted ages of mice in the Buckley dataset (*r* = 0.48, *p* = 0.0104). (b) Microglia subtypes in the Buckley study were mapped to cells in the Keren‐Shaul dataset and used to define frequency features to train and test a model in the Buckley and Keren‐Shaul studies, respectively. Plots show the chronological versus the predicted ages of mice in the Buckley dataset (*r* = 0.98, *p* = 0.0005). (c) Cells from the Buckley and Hammond datasets were integrated with the Scanorama algorithm to correct for dataset‐specific batch effects and labeled by joint cluster and colored by age (days). Cluster 9 contains cells from older mice and corresponds to the Homeostatic 1 microglia subtype. (d) Scanorama‐computed embeddings of cells in each pair of integrated datasets (with Hammond/Buckley on the left and Keren‐Shaul/Buckley on the right) were visualized in 2‐dimensional UMAP space.

After correcting cells in each dataset for dataset‐specific batch effects with the Scanorama algorithm (Hie et al. 2019), cells were clustered and visualized in two dimensions (Figure 5c,d). Clustering scanorama‐corrected cells across the Hammond and Buckley datasets, Cluster 9 was comprised of cells from older mice across both the Hammond and Buckley datasets (Figure 5c) and corresponded to the homeostatic 1 microglia subtype, based on the expression of *Hexb*, *Csf1r*, *Tgfbr1*, and *Selplg*. Figure 5a shows the predicted ages of each mouse in the Buckley dataset, trained by mapping Hammond subtype (e.g., cluster) labels to Buckley cells through a *k*‐nearest neighbor classifier to define frequency features (*r* = 0.48, *p* = 0.0104 between chronological and predicted age). Given that the mice in the Buckley study are generally older than those in the Hammond study, the moderate correlation suggests generalizability of the key aging‐linked frequency signals between the Hammond and Buckley datasets.

Leveraging mice from the Buckley and Keren‐Shaul datasets, which cover similar age ranges, we next mapped Buckley microglia subtype labels onto Keren‐Shaul cells to construct frequency features (Figure 5, panels b and d). The Buckley‐trained model extended seamlessly to the Keren‐Shaul dataset (*r* = 0.98, *p =* 0.0005) (Figure 5b), suggesting adequate identification of conserved microglia subtypes with frequencies that robustly change with age.

### 2.8 Identifying Key Dynamic Modules of Genes Changing With the Aging Trajectory

**FIGURE 6:**
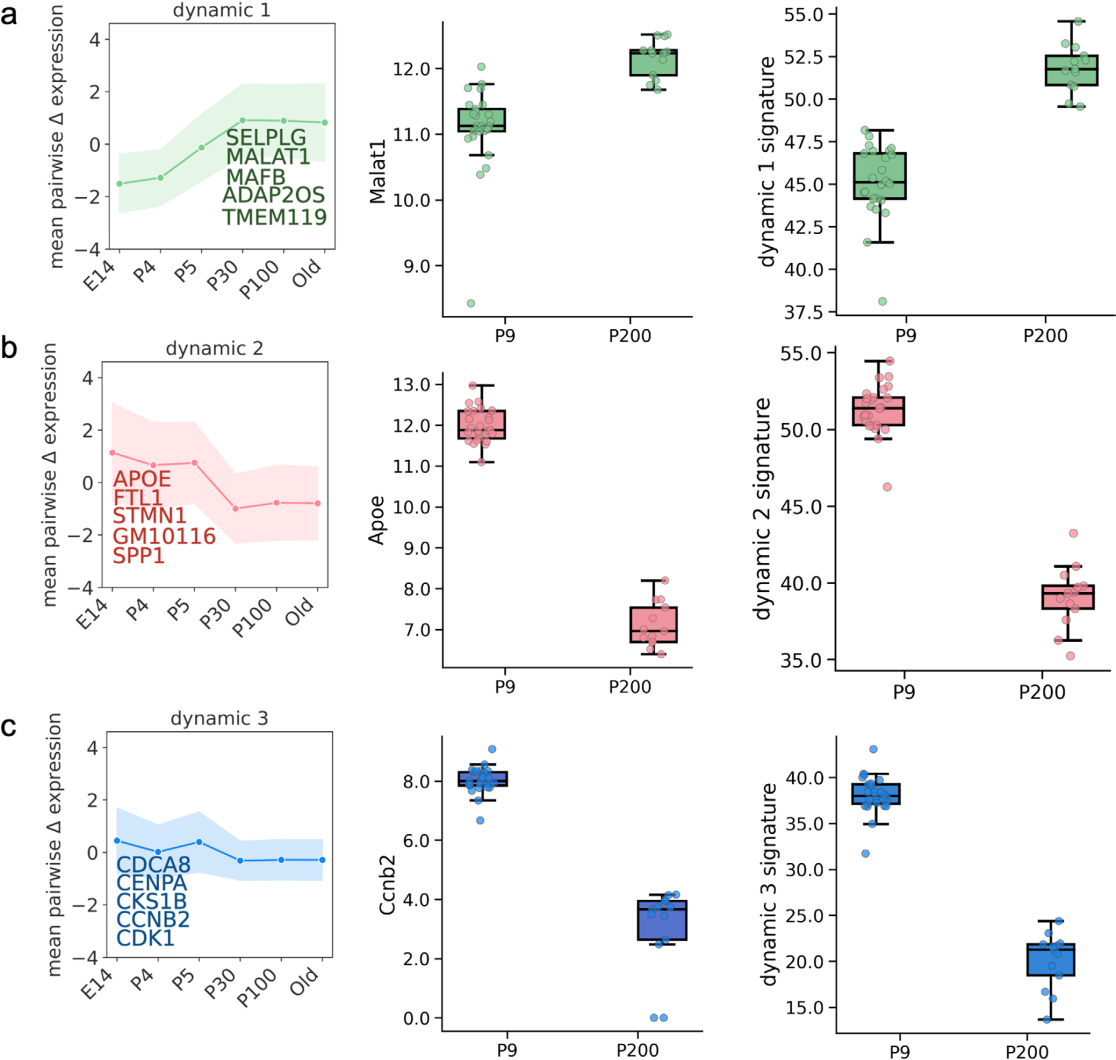
Modules of genes with characteristic dynamic changes in expression during aging are conserved across datasets. DELVE was used to identify modules of genes with common patterns of increase (a) or decrease (b, c) across the aging continuum in the Hammond dataset and applied to an independent bulk RNA‐seq dataset. The top five genes for each module are highlighted. The left panel shows the average expression pattern across the mouse lifespan for five representative genes in each particular module. The middle panels show the distribution of expression patterns for a prominent, representative gene (*Malat1*, *Apoe*, and *Ccnb2*) in dynamic modules 1, 2, and 3, respectively in P9 and P200 mice. The right panels show distributions of aggregate (e.g., summed) gene expressions of the indicated top five genes in each module in P9 and P200 mice.

We next sought to identify the groups of genes that underlie the changes in the subtype frequencies driving the microglia aging clocks. To this end, we used the DELVE algorithm (Ranek et al. 2024) to uncover groups of genes changing dynamically (i.e., systematic increase or decrease in expression) during aging. Applying DELVE in the Hammond dataset identified four dynamic gene modules with prominent changes across the aging continuum. As shown in Figure 6 left, key modules and their respective top‐scoring genes exhibited characteristic monotonic increases (Figure 6a) or decreases (Figure 6b) in expression over the aging continuum. DELVE also identified three dynamic gene modules in the Buckley dataset (Figure S12). Inferred gene modules uncovered through DELVE in the Buckley and Hammond datasets are visualized in Figure S13 and are provided as Supporting Information tables entitled DELVE_modules_Buckley.csv and DELVE_modules_Hammond.csv. As a further complementary validation of the key aging‐dependent dynamic transcriptomic signatures, we then examined the expression of representative genes in each module (Figure 6 middle) in an independent bulk RNA‐sequencing dataset generated by Reemst et al. (2022) (hereinafter denoted as the Reemst dataset), profiling microglia from young mice (P9, 9 days of age) and older mice (P200, 200 days of age). Strikingly, the expression patterns of genes uncovered in modules in the Hammond dataset tracked similarly in the Reemst dataset (Figure 6 middle), suggesting the trends are robust and generalize between single‐cell and bulk datasets. Similarly, composite expression scores obtained by summing the expression of the top five highlighted genes in each module in each mouse are shown in Figure 6 right and show consistent patterns of increase or decrease, according to the DELVE modules and could serve as bulk‐level aging signatures.

Analysis with gene ontology run with the g:profiler tool (Kolberg et al. 2023) revealed distinct biological processes in each DELVE module identified in the Hammond dataset. Gene ontology categories enriched in dynamic module 1 included cytoskeleton organization (GO: 0007010), regulation of monocyte differentiation (GO:0045655), and lipopolysaccharide immune receptor activity (GO:0001875) (Figure S2). Dynamic module 2 was enriched for generation of precursor metabolites and energy (GO: 0006091), detoxification (GO:0098754), ATP:ADP antiporter activity (GO:0005471), and organonitrogen compound metabolic process (GO:1901564) (Figure S3). Dynamic module 3 was enriched for iron–sulfur cluster binding (GO:0051536), mitotic cell cycle (GO:0000278), and ubiquitin protein ligase binding (GO:0031625) (Figure S4).

### 2.9 Investigating Sex Differences

Given previous studies reporting sex differences in transcriptomic signatures in microglia during aging (Hanamsagar et al. 2017; Li et al. 2023; Kang et al. 2024), we investigated frequency differences of microglia subtypes between male and female mice. The Buckley dataset contained only male mice, and the Hammond dataset contained significantly more males than females and few mice per age group, making it difficult to statistically test for sex differences. Using a bulk RNA‐seq dataset profiling male and female mice at ages E18, P4, P14, and P60 (Hanamsagar et al. 2017) (denoted hereinafter as the Hanamsagar dataset), we used the Cibersort algorithm (Chen et al. 2018) to deconvolve the bulk signatures into predicted frequencies across Hammond microglia subtypes. Our results identified clusters 11 and 13 to have similar patterns in frequency across lifespan between the Hammond (Figure S14a) and Hanamsagar (Figure S14b) datasets. Clusters 11 and 13 correspond to proliferating microglia and border‐associated macrophages (MAC), respectively (see differentially expressed genes visualized in Figure S14c). Despite prominent qualitative differences in frequencies of males and females observed in Cluster 11 at embryonic Day 18 (E18) and in Cluster 13 at 60 days of life (P60), there were no statistically significant differences between sexes (*p* = 0.2209 for cluster 11 and *p =* 0.889 for cluster 13) or significant age‐sex interactions (*p =* 0.0649 for cluster 11 and *p* = 0.587 for cluster 13) based on a two‐way ANOVA.

### 2.10 Extrapolating to Bulk RNA‐Seq Datasets With Environmental Inputs

We next tested if microglia aging clocks trained on scRNA‐seq data can be applied to independent bulk RNA‐seq datasets. As bulk datasets are more readily available, such applicability would present a powerful opportunity to exploit the unique insights gained through single‐cell profiling to a wider range of settings. It would further facilitate the use of single cell‐derived markers to dissect how bulk‐level gene expression programs relevant to aging are experimentally modulated by various environmental inputs. To address these questions, we leveraged the bulk RNA‐seq dataset of flow‐sorted microglia from the hippocampus of young (9 days of life) and aged (200 days of life) mice generated by Reemst et al. (2022). In addition to obtaining donors across lifespan, some mice in this study were exposed to early life stress (ELS) from 2 to 9 days of life in the form of limited bedding. The rationale for leveraging this experimental dataset is that, beyond extrapolating scRNA‐seq‐derived aging markers to bulk RNA‐seq data, it would also enable us to test how an environmental stimulus (ELS) modulates these markers.

**FIGURE 7:**
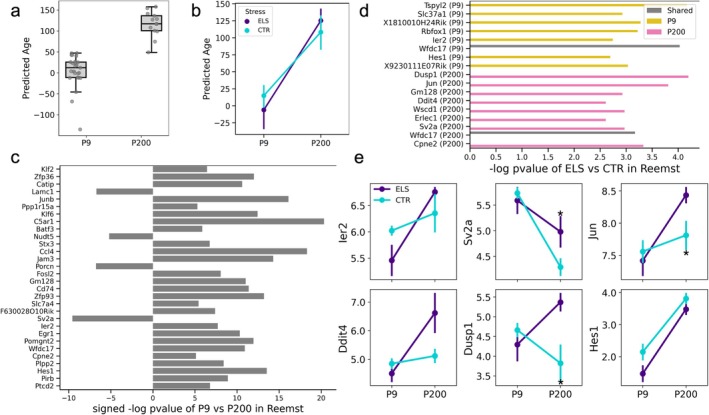
Aging clocks trained on scRNA‐seq are applicable to bulk RNA‐seq datasets with environmental inputs. (a) Boxplots show the distribution of predicted ages in P9 and P200 mice from both the ELS and control groups under the single‐cell to bulk‐level extrapolation approach. Predicted ages significantly differed between P9 and P200 mice (*p* = 1.69 × 10^−10^ under *t*‐test). (b) Samples were separated by stress condition at each age, and line plots visualize the trajectory in mean predicted age (points) at the model between P9 and P200 age groups (*p* = 0.144 for two‐way ANOVA to test the interaction between age and sex, linear regression *p* = 0.19 for ELS/CTR comparison for P9 and *p* = 0.37 for ELS/CTR comparison for P200). Error bars show the standard error of the mean. (c) Barplots visualize the key genes in the elastic net (EN) model trained on the Hammond dataset for predicting age that were also statistically significant (at *p* value < 10^−5^) for differentiating P9 and P200 mice across both control and ELS‐exposed conditions in the Reemst dataset. Barplots visualize the (signed) − log *p* value of each gene under a *t*‐test comparing its expression between P9 and P200 mice in the Reemst dataset. The sign (direction) of the bar indicates the sign of the coefficient of the gene in the EN model for age. (d) Barplots visualize −log *p* value (Wilcoxon test) of the ELS versus CTR comparison for the key genes prioritized by the elastic net (EN) model trained on the Hammond dataset. Genes were selected if they had a −log *p* value of ≥ 2.5 at ages P9 (gold), P200 (pink) or in both conditions (gray). (e) Line plots visualize expression patterns of key stress‐responsive genes, between P9 and P200 mice, separated by ELS (CTR) colored in purple (teal). Highlighted genes with differences between ELS and CTR groups include *Ier2* (*p* = 0.064 at P9), *Sv2a* (*p* = 0.05 at P200), *Jun* (*p* = 0.02 at P200), *Ddit4* (*p* = 0.07 at P200), *Dusp1* (*p* = 0.015 at P200), and *Hes1* (*p* = 0.067 at P9 and *p* = 0.115 at P200). Statistically significant expression differences between ELS and CTR mice (*p* ≤ 0.05) under a *t*‐test are denoted with an asterisk (*).

To assess the bulk‐level applicability of scRNA‐seq‐trained microglia clocks, we extracted the pseudobulk features from the PBHVG aging clock, which was trained earlier in the Hammond dataset, for the 2845 genes that were also measured in the Reemst dataset. To translate such pseudobulk features into a data structure similar to bulk RNA‐seq data with one expression value for each gene per sample, we summed each gene's per‐cluster composite value across all clusters. These per‐sample feature vectors of gene expression measurements were then used to train an Elastic Net (EN) regression model for age in the Hammond dataset (see Section 4 for details). When applied to the Reemst dataset, this single‐cell‐trained aging clock resulted in predicted ages that differed significantly between P9 and P200 mice across the control and ELS‐exposed groups (*p* = 1.69 × 10^−10^ under *t*‐test) (Figure 7a). We also tested whether ELS exposure impacted the aging clock. While there were no statistically significant differences between exposure groups and no statistically significant interaction between age and sex (*p* = 0.144) (Figure 7b), we noted an intriguing pattern whereby ELS resulted in lower predicted age in P9 (*p* = 0.19) but higher predicted age in P200 as compared to the control (CTR) condition (*p* = 0.37).

As a last step, we examined whether key genes identified by the PBHVG model to be implicated in aging also differentiate early life stress (ELS) and control (CTR) groups. This analysis revealed several transcriptomic signatures that are common between aging and stress exposure. We specifically considered the subset of genes with a coefficient with an absolute value ≥ 1.2 in the Hammond‐trained EN model (implying age relevance) and a −log *p* value ≥ 5 upon testing between P9 and P200 mice across both the control and stress‐exposed conditions in the Reemst dataset. These results identified conserved and distinct transcriptomic signatures between P9 and P200 mice, with key genes including *Sv2a*, *Lamc1*, *C5ar1*, *Egr1*, *Ccl4*, *Hes1*, *Batf3*, and *Zfp93* (Figure 7c). In addition, ELS exposure resulted in distinct gene expression differences between ELS and CTR groups in each age group. P9 mice exposed to ELS showed statistically significant differences in the expression of *Wfdc17* and *Tspyl2* (*p* < 0.05), whereas P200 mice exposed to ELS showed upregulation of *Dusp1* and *Jun* (*p* < 0.05), *Ddit4* (*p* = 0.07), and *Gm128* (*p* = 0.053) (Figure 7d). The dynamic patterns of these stress‐driven effects are visualized across the age groups in Figure 7e. Given that no stress‐related information was used to train the model, it is intriguing that some of the key genes with expression patterns that change during aging also exhibit expression differences in response to early life stress.

To further demonstrate the applicability of the single‐cell to bulk extrapolation procedure through pseudobulk features, we used the Hammond‐trained model to predict the ages of the embryonic Day 18 (E18), Day 4 (P4), Day 14 (P14), and Day 60 (P60) mice in the Hanamsagar (Hanamsagar et al. 2017) dataset (Figure S16). These results show the increase in predicted age over the lifespan and statistically significant pairwise differences between adjacent age groups, including E18 and P4 (*p* = 0.0068) and P14 and P60 (*p* = 0.047), overall supporting the applicability of the single‐cell trained clock.

## 3 Discussion

Biological aging clocks have been traditionally developed through bulk‐level analyses (Horvath 2013; Hannum et al. 2013; Lu et al. 2019; Levine et al. 2018; Belsky et al. 2022; Vijayakumar and Cho 2022), but recent evidence has highlighted the importance of gaining cell‐type and single cell‐level insights into the aging process (Horvath et al. 2015, 2014; Sonawane et al. 2017; Kabacik et al. 2022; Coninx et al. 2020; Dulken et al. 2019). Interrogating the transcriptome of key immune cell types, such as microglia (Aloisi 2001; Colonna and Butovsky 2017; Koo and Wohleb 2021; Chen et al. 2023; Bolte and Lukens 2021), may hold particular promise for gaining such insights. Leveraging scRNA‐seq datasets and state‐of‐the‐art computational methods, we generated robust microglia‐derived aging markers, compared marker performance across different featurization approaches, identified microglia genetic programs that change dynamically with aging, and showed that single‐cell insights extrapolate to bulk RNA‐seq data.

Rigorous testing of classification and regression‐based aging markers in the three single‐cell datasets highlighted ways in which signals across individual cells, samples, and datasets can be integrated to generate aging clocks. Leveraging a variety of such approaches, including frequency (Stanley et al. 2020; Bruggner et al. 2014), scLKME (Yi and Stanley 2023), our novel pseudobulk++ approach, and classical pseudobulk (Junttila et al. 2022), revealed intriguing tradeoffs between accuracy, biological interpretability, and efficiency. Frequency and pseudobulk++ compute compact, information‐rich features per microglia subtype, capturing the extent to which their abundances and gene expression patterns correlate with age. Frequency and pseudobulk++ proved to be top performers in age classification in the Hammond and Kracht datasets, and among the top two performing methods in regression‐based aging clocks in the Buckley and Hammond datasets that have samples from donors across a wide age range. In contrast, scLKME and classical pseudobulk produce higher‐dimensional feature representations, which inevitably pose larger demands on computation time and memory, but may encode more nuanced information that improves prediction in particular settings. For example, in the Buckley dataset, scLKME had significantly higher accuracy as an age classifier than the other methods, suggesting that the higher‐dimensional feature space was advantageous in this setting. Similarly, PBHVG and scLKME may be more accurate as aging clocks in challenging prediction tasks with limited age ranges, such as in the Kracht dataset. Overall, our results suggest that high‐dimensional featurization approaches (scLKME and PBHVG) are ideal for capturing dynamics in development where changes happen within a compressed timescale and with a potentially smaller effect size. In contrast, lower‐dimensional frequency‐based feature representations (e.g., frequency and PB++) are sufficient in aging studies where changes may occur over a more extended age range and at a larger scale. Practically, given that the datasets in the study were limited in the number of donors and sparsely sampled across the lifespan, future work on larger datasets could implement clocks through more complex nonlinear models to potentially enable more robust prediction. As our study only examines unsupervised approaches, there are promising opportunities to further explore how supervised, learning‐based featurization (Yi and Stanley 2021; Arvaniti and Claassen 2017), and their variants incorporating additional donor‐level covariates (Chen et al. 2024) can be applied to create more information‐rich encodings of single‐cell profiles.

Prior work has supported central roles for microglia in the neuroinflammation associated with aging (Sierra et al. 2007; Mosher and Wyss‐Coray 2014), neurodegenerative states (Prater et al. 2023; Song and Colonna 2018; Chen et al. 2023), and diverse neuropsychiatric disorders (Singhal and Baune 2017; Prinz and Priller 2014). However, the specific microglia subtypes and biological processes underlying these roles are unclear. Notably, our main analyses included diverse datasets derived from both the entire brain (Hammond) and the subventricular zone (Buckley). Leveraging, comparing, and combining such diverse datasets was in part driven by dataset accessibility and imposes certain limitations, such as the potential to miss important features that would perform more robustly across datasets from the same brain regions. Nevertheless, microglia show substantial heterogeneity across brain regions (Tan et al. 2020; Barko et al. 2022), and we argue that combining such diverse datasets can also uncover shared computational principles for modeling age and yield more generalizable markers of aging. Indeed, our unsupervised machine learning analyses, applied across three independent datasets, indicate dynamic age‐ and development‐related changes in the frequency of microglia subtypes, which are marked by biologically relevant key signatures. For example, *Apoe* encodes an apolipoprotein that has a well‐established role in neurodegeneration and dementia (Kloske et al. 2024), and *Malat1* codes for a long noncoding RNA that is dysregulated in immune cell subtypes in association with aging and frailty (Luo et al. 2022). Notably, *Malat1* exhibited opposite changes with age across different cell clusters, underscoring the importance of scRNA‐seq data, since it is in these cases that performing bulk analyses only would carry the risk of losing important biological signals. Moreover, some of these differences in direction are observed between aging and development datasets, suggesting potentially dynamic gene expression changes across the lifespan continuum. These findings corroborate and extend recent scRNA‐seq studies showing that transcriptional alterations during aging involve specific microglia subtypes and key genetic programs relevant to aging‐related brain phenotypes, including neural stem cell proliferation, neurodegeneration, and Alzheimer's disease (Hammond et al. 2019; Buckley et al. 2023; Ximerakis et al. 2019; Keren‐Shaul et al. 2017; Silvin et al. 2022). On the other hand, we also identify genes that show cluster‐specific changes only confined to fetal development, with notable examples including *Cx3cr1*, the gene encoding C‐X3‐C motif chemokine receptor 1, and *Csf1r*, the gene encoding colony‐stimulating factor 1 receptor. Applying dynamic gene module identification (with DELVE (Ranek et al. 2024)) and ontology analyses further identified gene modules enriched for distinct biological processes, including cytoskeleton organization, immune cell differentiation, metabolism‐related processes, cell cycle regulation, and ubiquitination. This framework can be applied in future aging studies to leverage novel transcriptomic datasets profiling microglia, such as human donors profiled in the PsychAD consortium (Lee et al. 2024; He et al. 2024), to more extensively characterize the role of microglia in brain aging and neuropsychiatric disorders.

Our results also indicate that single cell‐trained aging clocks are applicable to bulk‐level data. More specifically, we found that our pseudobulk clock trained from scRNA‐seq data from the entire brain (Hammond) robustly predicted age in an independent bulk RNA‐seq dataset derived from the hippocampus (Reemst), supporting that scRNA‐seq‐derived microglia aging markers extrapolate and generalize across modalities and brain regions. Consistent with prior work showing convergent genomic effects of stress and aging (Zannas et al. 2019; Wolf et al. 2019; Matosin et al. 2023), we further found that ELS significantly influenced the bulk‐level expression of several top genes comprising the pseudobulk clock, with effect magnitudes differing between age groups. Notable examples include the genes encoding the master transcription factor AP‐1 (*Jun*) (Jia et al. 2023) and the key innate immunity regulator *Dusp1* (Abraham and Clark 2006). This suggests that single‐cell‐derived transcriptomic signatures of aging are applicable to bulk data and modulated by environmental input. If confirmed by future studies, such single‐cell to bulk‐level applicability presents powerful opportunities to exploit the unique biological insights gained by single‐cell datasets to a wider range of settings, spanning large‐scale human cohorts and more nuanced experimental systems. Future studies may also uncover the potential of applying scRNA‐seq‐derived microglia aging clocks to bulk RNA‐seq datasets to predict lifespan and health span.

In summary, the present study builds on the highly promising biological aging research by leveraging scRNA‐seq data and state‐of‐the‐art machine learning methods to identify robust microglia aging clocks and dynamic cell‐type‐specific genetic programs. Such single‐cell‐derived and cell type‐specific clocks can yield unique insights into brain aging, ultimately promoting interventions that beneficially modulate health and disease trajectories.

## 4 Methods

### 4.1 Data Acquisition and Preprocessing

The Hammond (Hammond et al. 2019) (GSE121654), Buckley (Buckley et al. 2023) (GSE196364), and Kracht (Kracht et al. 2020) (GSE141862) scRNA‐seq datasets were downloaded from Gene Expression omnibus (GEO). Associated accession numbers are indicated in parentheses. Note that in the Buckley dataset, we converted the Seurat multi_integrated_seurat_Dec2020.rds object available in Zenodo (https://zenodo.org/records/7145399) as mentioned in Ref. Buckley et al. (2023) to an AnnData object for our experiments. The details of each dataset are described below.

#### 4.1.1 Hammond Mouse Lifespan

The Hammond study profiled mice throughout the lifespan (Hammond et al. 2019), with samples collected from the whole brain at embryonic day 14.5 (E14.5) and postnatal days 4 (P4), 5 (P5), 30 (P30), 100 (P100), and 540 (P540). Our analysis included a total of 34,700 microglia isolated by fluorescence‐activated cell sorting (FACS) in 47 total samples from 41 unique donors.

#### 4.1.2 Buckley Mouse Aging

The Buckley mouse aging study (Buckley et al. 2023) is comprised of *N* = 28 mice sampled at different ages between 3.3 and 29 months. Our analysis included a total of 3,844 cells. In the original study, cells extracted from the subventricular zone (SVZ) neurogenic region were profiled with scRNA‐seq, but we selected microglia according to marker genes indicated in the original study, including *C1qb*, *C1qb*, *Cst3*, *C1qc*, *Ctss*, *Hexb*, *Fcer1g*, *Trem2*, and *Tyrobp*.

#### 4.1.3 Kracht Fetal Development

The Kracht fetal development study (Kracht et al. 2020) profiled microglia that were isolated by FACS in postmortem CNS tissue from 20 aborted human fetuses between 9 and 18 gestational weeks, comprising 223 sample batches and 16,313 cells.

We performed downsampling in datasets as necessary to increase computational efficiency. Within a dataset, for a given sample ***i***, $\max\left\{750,\text{total number of cells in sample}\;i\right\}$ were randomly selected. Cells across samples were then concatenated into an annData object for use with single‐cell preprocessing tools in Scanpy. In each sample, we performed Counts per million (CPM) normalization by normalizing each cell by the total number of counts across all genes, using the function scanpy.pp.normalize_total with a target sum of 1e6. We then performed $\log\left(1+x\right)$ transformation for all counts. In the Hammond and Buckley datasets, the 3,000 most highly variable genes were retained. In the Kracht dataset, there were only 1,685 measured genes, and hence we did not do any additional highly variable gene filtering.

### 4.2 Single‐Cell and Bulk RNA‐seq Datasets Used for Validation

#### 4.2.1 Keren‐Shaul Single‐Cell Dataset

We obtained six healthy aging mice from the Keren‐Shaul study (Keren‐Shaul et al. 2017) at 49 and 600 days of life. Samples were obtained by sorting immune cells from whole brains. Our analysis considered a total of 1,772 cells across the *N* = 6 donors.

#### 4.2.2 Bulk RNA‐Sequencing Datasets

##### 4.2.2.1 Reemst Bulk RNA‐Sequencing Dataset

The Reemst bulk RNA‐sequencing dataset (Reemst et al. 2022) was downloaded from GEO (accession number GSE207067). This dataset profiles mice at 9 (P9) and 200 (P200) days of life after exposure to early life stress (ELS) through limited bedding from Days 2 to 9 of life. All gene expression measurements were $\log\left(1+x\right)$ transformed. Although mice in this study were also either untreated or injected with lipopolysaccharide (LPS), our analyses did not consider any sample from a mouse that had received LPS, as this is a potent immune stimulus that significantly affects microglia function.

##### 4.2.2.2 Hanamsagar Bulk RNA‐Sequencing Dataset

The Hanamsagar bulk RNA‐sequencing dataset profiles microglia in the hippocampus at Embryonic Day 18 (E18), and 4 (P4), 14 (P14), and 60 (P60) days of life. Although mice in this study were also untreated or injected with lipopolysaccharide (LPS), our analyses did not consider LPS‐treated samples due to the profound effects of LPS on microglia function. All gene expression measurements were $\log\left(1+x\right)$ transformed. We used the Cibersort algorithm implemented via an online web‐tool (https://cibersortx.stanford.edu/) to deconvolve the Hanamsagar into frequencies of microglia subtypes identified from the Hammond dataset.

### 4.3 Notation and Preliminaries

We define *X as* a single‐cell dataset over *N* profiled samples comprised of Ncell×gene matrices as $X={\left\{X_{i}\right\}}_{i=1}^{N}$. Here, $X_{i}\in R^{c_{i}\times p}$ gives the data matrix of p transcriptomic features measured across $c_{i}$ cells in a particular sample, *i*. We furthermore define a vector of per‐sample ages, $y\in R^{N}$, such that the *i*‐th element, $y_{i}$, gives the age of profiled sample *i*. Moreover, our task is to employ a robust featurization strategy to create a per‐sample microglia transcriptomic summary given by *d* features, $s_{i}\in R^{d}$, such that some model, $f\left(\cdot \right)$ can accurately model age so that $\sum_{i=1}^{N}\left[f\left(s_{i}\right)-y_{i}\right]$ is as small as possible.

### 4.4 Defining Cell Types Through Unsupervised Clustering

The partitioning of cells into clusters forms the backbone of many of the featurization approaches, which engineer sample‐level features based on clusters or uncovered cell types. To preprocess cells for input to clustering algorithms, we used scanpy to first represent each cell in terms of its top 40 principal components (PCs = 40) and built a *k*‐nearest neighbor graph by connecting each cell to its 10 nearest neighbors. To partition single cells into salient cell types with common gene expression programs, we used Leiden clustering (Traag et al. 2019) on constructed graph representation of the data, with resolution parameter, *γ* = 1. This algorithm partitions cells in each sample, $X_{i}$ into *K* populations. Note that this default resolution parameter, γ will produce different numbers of clusters per dataset, depending on the total number of cells and the extent of heterogeneity across cells.

### 4.5 Featurization Strategies

#### 4.5.1 Frequency

Given a partitioning of cells in a given sample, $X_{i}$, into 1 of *K* populations, we engineer a frequency feature vector, $f^{i}$, for sample *i*, such that the *m*‐th entry, $f_{m}^{i}$, encodes the proportion of cells in $X_{i}$ that were assigned to cluster *m*. This implies that the frequencies sum to 1 with $\sum_{m}f_{m}^{i}=1$.

#### 4.5.2 Classical Pseudobulk (PBHVG)

Classical psuedobulk is a standard formulation for compressing aggregate information across cells into single per‐sample feature vectors. Here, we compute classical pseudobulk featuresbased only on the most highly variable genes in each dataset For a given sample, $X_{i}$, the objective is to compute the summed expressions of each gene across all cells within a particular cluster, *m*. This process is repeated across the *K* clusters. So, we can define a pseudobulk feature vector for sample, *i* as $p^{i}$ by concatenating their per‐each‐cluster feature representations ($g_{m}^{i}$ for cluster *m* in sample *i*) as,

$$p^{i}=[g_{1}^{i}|g_{2}^{i}|\ldots |g_{K}^{i}]=[g_{11}^{i},g_{12}^{i},\ldots ,g_{1p}^{i}|g_{21}^{i},g_{22}^{i},\ldots ,g_{2p}^{i}|\ldots |g_{K1}^{i},g_{K2}^{i},\ldots ,g_{Kp}^{i}]$$

Here, for a dataset with *p* genes, each $g_{m}^{i}\in R^{p}$ is a vector of aggregated expressions over the *p* measured genes from all cells in sample *i* that have been assigned to cluster *m*. We chose sum as our aggregation technique so that a particular value, $g_{\mathrm{qm}}^{i}$ gives the sum of expression of gene *q* across all cells in cluster *m* in sample *i*.

#### 4.5.3 Pseudobulk++

Pseudobulk++ (PB++) is a novel featurization strategy introduced here, which is a parsimonious combination of frequency and classical pseudobulk. For each sample, this method computes a score for each cluster reflecting both the frequency of the sample's cells for that cluster and the extent to which the sample's cells in that cluster express key genes.

First, we perform a differential expression test in each cluster, which implements a *cluster* vs. *rest* comparison in each of the *K* clusters. We then rank genes by *p* value from most to least significant, such that the genes at the top of the list are those that are significantly differentially expressed through prominent upregulation or downregulation in at least one cluster. We then extract the top *G* genes, in terms of their *p* values (and z‐scores) across clusters to form the vector, *f*, such that $f=\left[f_{1},\ldots ,f_{G}\right]\in R^{G}$. Here, $f_{q}$ gives the absolute value of the z‐score for gene *q*, reflecting its strength of differential expression (either significantly up or down regulated) in the one versus rest differential expression test in at least one of the clusters. To implement differential expression tests, we used get.rank_genes_groups in scanpy. In practice, we found the method works well for the top 25 or top 50 differentially expressed genes and their respective scores across all clusters to yield *G* = 25 and *G* = 50 for PB++25 and PB++50, respectively. Ultimately, the set of *G* genes will be treated as a common set of key genes used to compute a composite per‐cluster score for each sample.

For a particular sample, *i*, we extract a subset of their cell × gene matrix from $X_{i}$ as ${\tilde{X}}_{i}$ with ${\tilde{X}}_{\mathrm{im}}\in R^{c_{\mathrm{im}}}\times G$, which gives the expression of only the common top *G* genes across the *c*_*im*_ cells in cluster *m* in sample *i*. The feature value, ${\tilde{p}}_{\mathrm{im}}$ for cluster *m* in sample *i* is therefore computed as,

$${\tilde{p}}_{\mathrm{im}}={\left({\tilde{X}}_{\mathrm{im}}\times f\right)}^{T}1^{c_{\mathrm{im}}}$$

Here, $1^{c_{\mathrm{im}}}$ is notation for the vector of length *c*_*im*_ (e.g., the number of cells from sample *i* in cluster *m* and provides an operator to sum the re‐weighted values of cells attained by ${\left({\tilde{X}}_{\mathrm{im}}\times f\right)}^{T}$). Moreover, the entire feature vector for sample *i* under the PB++ formulation can be expressed as,

$${\tilde{p}}_{i}=\left[{\tilde{p}}_{i1},\ldots ,p_{\mathrm{im}}\right]=\left[{\left({\tilde{X}}_{i1}\times f\right)}^{T}1^{c_{i1}},\ldots ,{\left({\tilde{X}}_{\mathrm{ik}}\times f\right)}^{T}1^{c_{\mathrm{iK}}}\right].$$

Ultimately, each ${\tilde{p}}_{i}\in R^{K}$ has only as many features as there are clusters.

#### 4.5.4 scLKME

The scLKME strategy was introduced in Ref. Yi and Stanley (2023). The premise of the algorithm is to choose a number of landmark cells across all profiled samples and to ultimately compute featurizations based on overall patterns of each cell's similarity with each landmark as gleaned through kernel evaluations. We chose *L* = 1500 landmark cells obtained through cell sketching (Baskaran et al. 2022) across all samples and with kernel, $\kappa \left(\cdot \right)$, as the radial basis function (RBF). Under this formulation, scLKME computes the featurization for sample *i*, $s_{i}\in R^{L}$ (for *L* = 1500) as,

$$s_{i}=\left[\hat{\mu }\left(x_{l_{1}}\right),\hat{\mu }\left(x_{l_{1}}\right),\ldots ,\hat{\mu }\left(x_{l_{L}}\right)\right].$$

Here, each $\hat{\mu }\left(x_{l_{j}}\right)$ computes the mean kernel evaluation over all $c_{i}$ cells and sample *i* and the landmark cell, *l*_*j*_ as,

$$\hat{\mu }\left({Χ}_{\ell_{j}}\right)=\frac{1}{c_{i}}\sum_{t=1}^{c_{i}}\kappa \left({Χ}_{t},{Χ}_{\ell_{j}}\right).$$

### 4.6 Age Classifiers

Age classifiers were trained to predict the ages of samples binned in discrete categories. The Hammond dataset already had discrete age categories, consisting of E14.5, P4, P5, P30, P100, and P540, so the classification problem was over six classes. In the Buckley dataset, we binned ages into young (< 4 months), adult (4 months ≤ age < 14 months), and old (≥ 14 months). The classification problem was therefore over three age categories. Finally, we formulated a binary classification problem in the Kracht dataset by separating samples into first (9–12 weeks) and second trimester (13–18 weeks), respectively.

Within a dataset, we applied a given featurization approach to each sample to obtain its vector encoding. These vector encodings were then given to a random forest classifier (implemented in ScikitLearn using RandomForestClassifier in Python) with 50 trees and the square root of the total number of features to find the best split. We used 200 randomized train/test splits of the data using 80% of the donors for training and the remaining 20% for testing to obtain a distribution of classification accuracies. Classification accuracies reflect the proportion of samples with correctly predicted labels in the dataset.

### 4.7 Aging Clocks

We used penalized linear regression with the Lasso penalty (implemented in ScikitLearn using linear_model.Lasso in Python) to predict sample ages, under each given featurization. Given a featurization of *N* samples into *d* features obtained for a dataset that results in a matrix, $D\in R^{N\times f}$ with a corresponding age response vector as $y\in R^{N}$, Lasso regression seeks to optimize a vector of per‐feature coefficients, $\beta \in R^{f}$ that minimize,

$$\frac{1}{2N}{\left\|y-\mathrm{D\beta}\right\|}_{2}^{2}+\lambda {\left\|\beta \right\|}_{1}.$$

We tuned *λ*, or the magnitude of the penalty applied to each coefficient, through cross‐validation on each training set. To train and test the models, we used a leave‐one‐out cross‐validation approach (LOOCV) by training the model on all but the one held‐out sample and predicting the age for the held‐out sample in each LOOCV iteration. The Buckley dataset only has one sample per donor, whereas the Kracht and Hammond datasets have multiple samples per donor, such that samples from a donor were kept together in the same train/test split in each LOOCV iteration.

We used Pearson correlation and fitted linear regression *p* value as the metrics of success to quantify how well the chronological and predicted ages correlated across samples (implemented with ScikitLearn).

### 4.8 Cross‐Dataset Evaluation of Clocks

To perform cross‐dataset integration and analysis, dataset‐specific batch effects were corrected with the Scanorama algorithm (Hie et al. 2019). Briefly, given a pair of datasets to be integrated, one dataset is chosen as the reference to correct expression measurements in the other datasets with respect to. For integration between the Hammond and Buckley datasets, the Hammond dataset was used as the reference. For integration between the Buckley and Keren‐Shaul datasets, the Buckley dataset was used as the reference.

We used a *k*‐nearest neighbor mapping approach implemented with the function scanpy.tl.ingest() in the scanpy package to map cluster labels between datasets. Common cluster labels between datasets were used to define frequency features across a common set of microglia subtypes between two datasets. In integrating the Hammond and Buckley datasets, Hammond subtypes were mapped to Buckley cells. In integrating the Buckley and Keren‐Shaul datasets, Buckley subtype labels were mapped to Keren‐Shaul cells. After mapping between a dataset pair, an Elastic Net model was trained based on one member of the dataset pair and evaluated on the other member of the dataset pair.

### 4.9 Extrapolating From Single‐Cell to Bulk

To generate a scRNA‐seq‐derived aging clock that could be applied to the Reemst bulk RNA‐seq dataset (Reemst et al. 2022), we converted the multiple cell × gene expression matrices in the single‐cell Hammond dataset (Hammond et al. 2019) into a sample × gene expression matrix, which has analogous structure to the bulk RNA‐seq data. To this end, we used a simple variation of the classical pseudobulk approach. As classical pseudobulk computes an aggregate expression measurement for each gene in each cluster (usually by computing a sum), we decided to simply compute aggregate expression of each gene across all cells (so, across all clusters) through sum (alternative aggregation methods explored in Figure S5). So, given the Hammond dataset, ${\left\{X_{i}\right\}}_{i=1}^{N}$ profiling cells across *N*_*h*_ samples with *c*_*i*_ cells measured per sample, we computed the sample × gene matrix, $\tilde{Χ}\in R^{N_{h}\times G}$ for the dataset such that a given entry ${\tilde{Χ}}_{\mathrm{iq}}$ measuring the expression of gene *q* in sample *i* is computed as the sum of column *q* (gene *q*) over all cells as,

$${\tilde{X}}_{\mathrm{iq}}=\sum_{i=1}^{c_{i}}X_{\mathrm{iq}}$$

We consider only the *G* genes that are also measured in the Reemst Bulk RNA‐sequencing dataset profiling *N*_*r*_ samples, which produced a data matrix of $B\in R^{N_{r}\times G}$.

Using $\tilde{Χ}$ we trained an Elastic Net linear regression model to find per‐gene coefficients $\beta \in R^{G}$ to optimally predict ages in the Hammond dataset given by $y_{h}\in R^{N_{h}}$ that minimize,

$$\frac{1}{2N_{h}}{\left\|y_{h}-\tilde{Χ}\beta \right\|}_{2}^{2}+\left(\alpha \times l1\_\text{ratio}\right){\left\|\beta \right\|}_{1}+\left(0.5\times \alpha \times \left(1-l1\_\text{ratio}\right)\right){\left\|\beta \right\|}_{2}^{2}.$$

The model is trained using linear_model.ElasticNet in Scikit learn with default parameter values of α = 1 and *l*1_ratio = 0.5.

The ultimate per‐gene coefficients encoded in *β* were ultimately used to predict the ages ($y_{N_{r}}$) of samples in the Reemst dataset as,

$$y_{r}=B\times \beta .$$

### 4.10 DELVE Implementation to Identify Gene Modules With Common Genetic Patterns

We applied the DELVE algorithm (Ranek et al. 2024) to the Hammond dataset to reveal dynamic genetic programs in microglia in an unsupervised manner. We uncovered five dynamic modules with DELVE (using parameter *n*_clusters = 5) and otherwise default parameters.

## Author Contributions

N.S. and A.S.Z. conceptualized the study and designed experiments. Featurization techniques were developed and implemented by N.S., A.S.Z., L.D., and Y.‐C.H. Datasets were preprocessed by L.D., Y.‐C.H., and S.J. Data were analyzed by all authors. N.S., A.S.Z., and L.D. wrote the paper with input from all authors.

## Conflicts of Interest

The authors declare no conflicts of interest.

## Supporting information


Data S1.

## Data Availability

All featurization strategies and tutorials for reproducing results are available in github https://github.com/CompCy‐lab/microglia‐aging‐clock. Processed scRNA‐seq datasets are available in anndata format in Zenodo (DOI: https://doi.org/10.5281/zenodo.12811383).

## References

[acel70095-bib-0001] Abraham, S. , and A. Clark . 2006. “Dual‐Specificity Phosphatase 1: A Critical Regulator of Innate Immune Responses.” Biochemical Society Transactions 34, no. 6: 1018–1023. 17073741 10.1042/BST0341018

[acel70095-bib-0002] Aloisi, F. 2001. “Immune Function of Microglia.” Glia 36, no. 2: 165–179. 11596125 10.1002/glia.1106

[acel70095-bib-0003] Arvaniti, E. , and M. Claassen . 2017. “Sensitive Detection of Rare Disease‐Associated Cell Subsets via Representation Learning.” Nature Communications 8, no. 1: 1–10. 10.1038/ncomms14825 PMC5384229 28382969

[acel70095-bib-0004] Barko, K. , M. Shelton , X. Xue , et al. 2022. “Brain Region‐ and Sex‐Specific Transcriptional Profiles of Microglia.” Frontiers in Psychiatry 13: 945548. 36090351 10.3389/fpsyt.2022.945548 PMC9448907

[acel70095-bib-0005] Baskaran, V. A. , J. Ranek , S. Shan , N. Stanley , and J. B. Oliva . 2022. “Distribution‐Based Sketching of Single‐Cell Samples.” In: Proceedings of the 13th ACM International Conference on Bioinformatics, Computational Biology and Health Informaticspp. 1–10.

[acel70095-bib-0006] Belsky, D. W. , A. Caspi , D. L. Corcoran , et al. 2022. “DunedinPace, a DNA Methylation Biomarker of the Pace of Aging.” eLife 11: 73420. 10.7554/eLife.73420 PMC8853656 35029144

[acel70095-bib-0007] Biltz, R. G. , C. M. Sawicki , J. F. Sheridan , and J. P. Godbout . 2022. “The Neuroimmunology of Social‐Stress‐Induced Sensitization.” Nature Immunology 23, no. 11: 1527–1535. 36369271 10.1038/s41590-022-01321-z PMC10000282

[acel70095-bib-0008] Bolte, A. C. , and J. R. Lukens . 2021. “Neuroimmune Cleanup Crews in Brain Injury.” Trends in Immunology 42, no. 6: 480–494. 33941486 10.1016/j.it.2021.04.003 PMC8165004

[acel70095-bib-0009] Bruggner, R. V. , B. Bodenmiller , D. L. Dill , R. J. Tibshirani , and G. P. Nolan . 2014. “Automated Identification of Stratifying Signatures in Cellular Subpopulations.” Proceedings of the National Academy of Sciences of the United States of America 111, no. 26: 2770–2777. 24979804 10.1073/pnas.1408792111 PMC4084463

[acel70095-bib-0010] Buckley, M. T. , E. D. Sun , B. M. George , et al. 2023. “Cell‐Type‐Specific Aging Clocks to Quantify Aging and Rejuvenation in Neurogenic Regions of the Brain.” Nature Aging 3, no. 1: 121–137. 37118510 10.1038/s43587-022-00335-4 PMC10154228

[acel70095-bib-0011] Chen, B. , M. S. Khodadoust , C. L. Liu , A. M. Newman , and A. A. Alizadeh . 2018. “Profiling Tumor Infiltrating Immune Cells With CIBERSORT.” Cancer Systems Biology 1711: 243–259. 10.1007/978-1-4939-7493-1_12 PMC5895181 29344893

[acel70095-bib-0012] Chen, C.‐J. , H. Yi , and N. Stanley . 2024. “Conditional Similarity Triplets Enable Covariate Informed Representations of Single‐Cell Data.” arXiv. 10.48550/arXiv.2406.08638. PMC11807331 39924480

[acel70095-bib-0013] Chen, X. , M. Firulyova , M. Manis , et al. 2023. “Microglia‐Mediated T Cell Infiltration Drives Neurodegeneration in Tauopathy.” Nature 615, no. 7953: 668–677. 36890231 10.1038/s41586-023-05788-0 PMC10258627

[acel70095-bib-0014] Colonna, M. , and O. Butovsky . 2017. “Microglia Function in the Central Nervous System During Health and Neurodegeneration.” Annual Review of Immunology 35, no. 1: 441–468. 10.1146/annurev-immunol-051116-052358 PMC8167938 28226226

[acel70095-bib-0015] Coninx, E. , Y. C. Chew , X. Yang , et al. 2020. “Hippocampal and Cortical Tissue‐Specific Epigenetic Clocks Indicate an Increased Epigenetic Age in a Mouse Model for Alzheimer's Disease.” Aging (Albany NY) 12, no. 20: 20817–20834. 33082299 10.18632/aging.104056 PMC7655172

[acel70095-bib-0016] Dulken, B. W. , M. T. Buckley , P. Navarro Negredo , et al. 2019. “Single‐Cell Analysis Reveals T Cell Infiltration in Old Neurogenic Niches.” Nature 571, no. 7764: 205–210. 31270459 10.1038/s41586-019-1362-5 PMC7111535

[acel70095-bib-0017] Fan, H. , M. Zhang , J. Wen , et al. 2024. “Microglia in Brain Aging: An Overview of Recent Basic Science and Clinical Research Developments.” Journal of Biomedical Research 38: 122–136. 38403286 10.7555/JBR.37.20220220 PMC11001587

[acel70095-bib-0018] Frigerio, C. S. , L. Wolfs , N. Fattorelli , et al. 2019. “The Major Risk Factors for Alzheimer's Disease: Age, Sex, and Genes Modulate the Microglia Response to Aβ Plaques.” Cell Reports 27: 1293–1306. 31018141 10.1016/j.celrep.2019.03.099 PMC7340153

[acel70095-bib-0019] Galatro, T. F. , I. R. Holtman , A. M. Lerario , et al. 2017. “Transcriptomic Analysis of Purified Human Cortical Microglia Reveals Age‐Associated Changes.” Nature Neuroscience 20: 1162–1171. 28671693 10.1038/nn.4597

[acel70095-bib-0020] Gerrits, E. , Y. Heng , E. W. Boddeke , and B. J. Eggen . 2020. “Transcriptional Profiling of Microglia; Current State of the Art and Future Perspectives.” Glia 68: 740–755. 31846124 10.1002/glia.23767 PMC7064956

[acel70095-bib-0021] Hammond, T. R. , C. Dufort , L. Dissing‐Olesen , et al. 2019. “Single‐Cell RNA Sequencing of Microglia Throughout the Mouse Lifespan and in the Injured Brain Reveals Complex Cell‐State Changes.” Immunity 50, no. 1: 253–271. 30471926 10.1016/j.immuni.2018.11.004 PMC6655561

[acel70095-bib-0022] Hanamsagar, R. , M. D. Alter , C. S. Block , H. Sullivan , J. L. Bolton , and S. D. Bilbo . 2017. “Generation of a Microglial Developmental Index in Mice and in Humans Reveals a Sex Difference in Maturation and Immune Reactivity.” Glia 65, no. 1: 1504–1520. 28618077 10.1002/glia.23176 PMC5540146

[acel70095-bib-0023] Hannum, G. , J. Guinney , L. Zhao , et al. 2013. “Genome‐Wide Methylation Profiles Reveal Quantitative Views of Human Aging Rates.” Molecular Cell 49, no. 2: 359–367. 23177740 10.1016/j.molcel.2012.10.016 PMC3780611

[acel70095-bib-0024] He, C. , A. Z. Li , K. H. Arachchilage , et al. 2024. “Phenotype Scoring of Population Scale Single‐Cell Data Dissects Alzheimer's Disease Complexity.” medRxiv. 10.1101/2024.11.01.24316586.

[acel70095-bib-0025] Hie, B. , B. Bryson , and B. Berger . 2019. “Efficient Integration of Heterogeneous Single‐Cell Transcriptomes Using Scanorama.” Nature Biotechnology 37: 685–691. 10.1038/s41587-019-0113-3 PMC6551256 31061482

[acel70095-bib-0026] Horvath, S. 2013. “DNA Methylation Age of Human Tissues and Cell Types.” Genome Biology 14: 1–20. 10.1186/gb-2013-14-10-r115 PMC4015143 24138928

[acel70095-bib-0027] Horvath, S. , W. Erhart , M. Brosch , et al. 2014. “Obesity Accelerates Epigenetic Aging of Human Liver.” Proceedings of the National Academy of Sciences of the United States of America 111, no. 43: 15538–15543. 25313081 10.1073/pnas.1412759111 PMC4217403

[acel70095-bib-0028] Horvath, S. , V. Mah , A. T. Lu , et al. 2015. “The Cerebellum Ages Slowly According to the Epigenetic Clock.” Aging (Albany NY) 7, no. 5: 294–306. 26000617 10.18632/aging.100742 PMC4468311

[acel70095-bib-0029] Horvath, S. , and K. Raj . 2018. “DNA Methylation‐Based Biomarkers and the Epigenetic Clock Theory of Ageing.” Nature Reviews Genetics 19, no. 6: 371–384. 10.1038/s41576-018-0004-3 29643443

[acel70095-bib-0030] Hu, Z. , B. S. Glicksberg , and A. J. Butte . 2019. “Robust Prediction of Clinical Outcomes Using Cytometry Data.” Bioinformatics 35, no. 7: 1197–1203. 30169745 10.1093/bioinformatics/bty768 PMC6449751

[acel70095-bib-0031] Jia, Q. , Y. Tan , Y. Li , Y. Wu , J. Wang , and F. Tang . 2023. “Jun‐Induced Super‐Enhancer RNA Forms R‐Loop to Promote Nasopharyngeal Carcinoma Metastasis.” Cell Death & Disease 14, no. 7: 459. 37479693 10.1038/s41419-023-05985-9 PMC10361959

[acel70095-bib-0032] Junttila, S. , J. Smolander , and L. L. Elo . 2022. “Benchmarking Methods for Detecting Differential States Between Conditions From Multi‐Subject Single‐Cell RNA‐Seq Data.” Briefings in Bioinformatics 23, no. 5: 286. 10.1093/bib/bbac286 PMC9487674 35880426

[acel70095-bib-0033] Kabacik, S. , D. Lowe , L. Fransen , et al. 2022. “The Relationship Between Epigenetic Age and the Hallmarks of Aging in Human Cells.” Nature Aging 2, no. 6: 484–493. 37034474 10.1038/s43587-022-00220-0 PMC10077971

[acel70095-bib-0034] Kang, S. , E. Y. Ko , A. E. Andrews , et al. 2024. “Microglia Undergo Sex‐Dimorphic Transcriptional and Metabolic Rewiring During Aging.” Journal of Neuroinflammation 21: 150. 38840206 10.1186/s12974-024-03130-7 PMC11155174

[acel70095-bib-0035] Keren‐Shaul, H. , A. Spinrad , A. Weiner , et al. 2017. “A Unique Microglia Type Associated With Restricting Development of Alzheimer's Disease.” Cell 169, no. 7: 1276–1290. 28602351 10.1016/j.cell.2017.05.018

[acel70095-bib-0036] Kloske, C. M. , M. E. Belloy , E. E. Blue , et al. 2024. “Advancements in APOE and Dementia Research: Highlights From the 2023 AAIC Advancements: APOE Conference.” Alzheimer's & Dementia 20, no. 9: 6590–6605. 10.1002/alz.13877. PMC11497726 39031528

[acel70095-bib-0037] Kolberg, L. , U. Raudvere , I. Kuzmin , P. Adler , J. Vilo , and H. Peterson . 2023. “G: Profiler—Interoperable Web Service for Functional Enrichment Analysis and Gene Identifier Mapping (2023 Update).” Nucleic Acids Research 51, no. W1: 207–212. 10.1093/nar/gkad347 PMC10320099 37144459

[acel70095-bib-0038] Koo, J. W. , and E. S. Wohleb . 2021. “How Stress Shapes Neuroimmune Function: Implications for the Neurobiology of Psychiatric Disorders.” Biological Psychiatry 90, no. 2: 74–84. 33485589 10.1016/j.biopsych.2020.11.007 PMC8126571

[acel70095-bib-0039] Korsunsky, I. , N. Millard , J. Fan , et al. 2019. “Fast, Sensitive and Accurate Integration of Single‐Cell Data With Harmony.” Nature Methods 16, no. 12: 1289–1296. 31740819 10.1038/s41592-019-0619-0 PMC6884693

[acel70095-bib-0040] Kracht, L. , M. Borggrewe , S. Eskandar , et al. 2020. “Human Fetal Microglia Acquire Homeostatic Immune‐Sensing Properties Early in Development.” Science 369, no. 6503: 530–537. 32732419 10.1126/science.aba5906

[acel70095-bib-0041] Lee, D. , M. Koutrouli , N. Y. Masse , et al. 2024. “Single‐Cell Atlas of Transcriptomic Vulnerability Across Multiple Neurodegenerative and Neuropsychiatric Diseases.” medRxiv. 10.1101/2024.10.31.24316513.

[acel70095-bib-0042] Levine, M. E. , A. T. Lu , A. Quach , et al. 2018. “An Epigenetic Biomarker of Aging for Lifespan and Healthspan.” Aging (Albany NY) 10, no. 4: 573–591. 29676998 10.18632/aging.101414 PMC5940111

[acel70095-bib-0043] Li, X. , Y. Li , Y. Jin , et al. 2023. “Transcriptional and Epigenetic Decoding of the Microglial Aging Process.” Nature Aging 3: 1288–1311. 37697166 10.1038/s43587-023-00479-x PMC10570141

[acel70095-bib-0044] Lu, A. T. , A. Quach , J. G. Wilson , et al. 2019. “DNA Methylation Grimage Strongly Predicts Lifespan and Healthspan.” Aging (Albany NY) 11, no. 2: 303–327. 30669119 10.18632/aging.101684 PMC6366976

[acel70095-bib-0045] Luo, O. J. , W. Lei , G. Zhu , et al. 2022. “Multidimensional Single‐Cell Analysis of Human Peripheral Blood Reveals Characteristic Features of the Immune System Landscape in Aging and Frailty.” Nature Aging 2, no. 4: 348–364. 37117750 10.1038/s43587-022-00198-9

[acel70095-bib-0046] Mao, S. , J. Su , L. Wang , X. Bo , C. Li , and H. Chen . 2023. “A Transcriptome‐Based Single‐Cell Biological Age Model and Resource for Tissue‐Specific Aging Measures.” Genome Research 33: 1381–1394. 37524436 10.1101/gr.277491.122 PMC10547252

[acel70095-bib-0047] Marschallinger, J. , T. Iram , M. Zardeneta , et al. 2020. “Lipid‐Droplet‐Accumulating Microglia Represent a Dysfunctional and Proinflammatory State in the Aging Brain.” Nature Neuroscience 23: 194–208. 31959936 10.1038/s41593-019-0566-1 PMC7595134

[acel70095-bib-0048] Martins‐Ferreira, R. , J. Calafell‐Segura , B. Leal , et al. 2025. “The Human Microglia Atlas (HuMicA) Unravels Changes in Disease‐Associated Microglia Subsets Across Neurodegenerative Conditions.” Nature Communications 16: 739. 10.1038/s41467-025-56124-1 PMC11739505 39820004

[acel70095-bib-0049] Masuda, T. , R. Sankowski , O. Staszewski , et al. 2019. “Spatial and Temporal Heterogeneity of Mouse and Human Microglia at Single‐Cell Resolution.” Nature 566: 388–392. 30760929 10.1038/s41586-019-0924-x

[acel70095-bib-0050] Mathys, H. , C. Adaikkan , F. Gao , et al. 2017. “Temporal Tracking of Microglia Activation in Neurodegeneration at Single‐Cell Resolution.” Cell Reports 21: 366–380. 29020624 10.1016/j.celrep.2017.09.039 PMC5642107

[acel70095-bib-0051] Matosin, N. , J. Arloth , D. Czamara , et al. 2023. “Associations of Psychiatric Disease and Ageing With fkbp5 Expression Converge on Superficial Layer Neurons of the Neocortex.” Acta Neuropathologica 145, no. 4: 439–459. 36729133 10.1007/s00401-023-02541-9 PMC10020280

[acel70095-bib-0052] Menze, B. H. , B. M. Kelm , R. Masuch , et al. 2009. “A Comparison of Random Forest and Its Gini Importance With Standard Chemometric Methods for the Feature Selection and Classification of Spectral Data.” BMC Bioinformatics 10: 1–16. 19591666 10.1186/1471-2105-10-213 PMC2724423

[acel70095-bib-0053] Meyer, D. H. , and B. Schumacher . 2021. “BiT Age: A Transcriptome‐Based Aging Clock Near the Theoretical Limit of Accuracy.” Aging Cell 20: e13320. 33656257 10.1111/acel.13320 PMC7963339

[acel70095-bib-0054] Mosher, K. I. , and T. Wyss‐Coray . 2014. “Microglial Dysfunction in Brain Aging and Alzheimer's Disease.” Biochemical Pharmacology 88, no. 4: 594–604. 24445162 10.1016/j.bcp.2014.01.008 PMC3972294

[acel70095-bib-0055] Mrdjen, D. , A. Pavlovic , F. J. Hartmann , et al. 2018. “High‐Dimensional Single‐Cell Mapping of Central Nervous System Immune Cells Reveals Distinct Myeloid Subsets in Health, Aging, and Disease.” Immunity 48: 380–395. 29426702 10.1016/j.immuni.2018.01.011

[acel70095-bib-0056] Prater, K. E. , K. J. Green , S. Mamde , et al. 2023. “Human Microglia Show Unique Transcriptional Changes in Alzheimer's Disease.” Nature Aging 3: 894–907. 37248328 10.1038/s43587-023-00424-y PMC10353942

[acel70095-bib-0057] Prinz, M. , and J. Priller . 2014. “Microglia and Brain Macrophages in the Molecular Age: From Origin to Neuropsychiatric Disease.” Nature Reviews Neuroscience 15, no. 5: 300–312. 24713688 10.1038/nrn3722

[acel70095-bib-0058] Ranek, J. S. , W. Stallaert , J. J. Milner , et al. 2024. “Delve: Feature Selection for Preserving Biological Trajectories in Single‐Cell Data.” Nature Communications 15, no. 1: 2765. 10.1038/s41467-024-46773-z PMC10980758 38553455

[acel70095-bib-0059] Reemst, K. , L. Kracht , J. M. Kotah , et al. 2022. “Early‐Life Stress Lastingly Impacts Microglial Transcriptome and Function Under Basal and Immune‐Challenged Conditions.” Translational Psychiatry 12, no. 1: 507. 36481769 10.1038/s41398-022-02265-6 PMC9731997

[acel70095-bib-0060] Shan, S. , V. A. Baskaran , H. Yi , J. Ranek , N. Stanley , and J. B. Oliva . 2022. “Transparent Single‐Cell Set Classification With Kernel Mean Embeddings.” In: Proceedings of the 13th ACM International Conference on Bioinformatics, Computational Biology and Health Informatics. pp. 1–10.

[acel70095-bib-0061] Shea, J. M. , and S. A. Villeda . 2024. “Microglia Aging in the Hippocampus Advances Through Intermediate States That Drive Inflammatory Activation and Cognitive Decline.” eLife 13: RP97671. 10.7554/eLife.97671 PMC12040317 40298588

[acel70095-bib-0062] Sierra, A. , A. C. Gottfried‐Blackmore , B. S. McEwen , and K. Bulloch . 2007. “Microglia Derived From Aging Mice Exhibit an Altered Inflammatory Profile.” Glia 55, no. 4: 412–424. 17203473 10.1002/glia.20468

[acel70095-bib-0063] Silvin, A. , S. Uderhardt , C. Piot , et al. 2022. “Dual Ontogeny of Disease‐Associated Microglia and Disease Inflammatory Macrophages in Aging and Neurodegeneration.” Immunity 55, no. 8: 1448–1465. 35931085 10.1016/j.immuni.2022.07.004

[acel70095-bib-0064] Singhal, G. , and B. T. Baune . 2017. “Microglia: An Interface Between the Loss of Neuroplasticity and Depression.” Frontiers in Cellular Neuroscience 11: 270. 28943841 10.3389/fncel.2017.00270 PMC5596091

[acel70095-bib-0065] Sonawane, A. R. , J. Platig , M. Fagny , et al. 2017. “Understanding Tissue‐Specific Gene Regulation.” Cell Reports 21, no. 4: 1077–1088. 10.1016/j.celrep.2017.10.001. 29069589 PMC5828531

[acel70095-bib-0066] Song, W. M. , and M. Colonna . 2018. “The Identity and Function of Microglia in Neurodegeneration.” Nature Immunology 19, no. 10: 1048–1058. 30250185 10.1038/s41590-018-0212-1

[acel70095-bib-0067] Stanley, N. , I. A. Stelzer , A. S. Tsai , et al. 2020. “Vopo Leverages Cellular Heterogeneity for Predictive Modeling of Single‐Cell Data.” Nature Communications 11, no. 1: 1–9. 10.1038/s41467-020-17569-8 PMC7385162 32719375

[acel70095-bib-0068] Tan, Y. L. , Y. Yuan , and L. Tian . 2020. “Microglial Regional Heterogeneity and Its Role in the Brain.” Molecular Psychiatry 25: 351–367. 31772305 10.1038/s41380-019-0609-8 PMC6974435

[acel70095-bib-0069] Traag, V. A. , L. Waltman , and N. J. Van Eck . 2019. “From Louvain to Leiden: Guaranteeing Well‐Connected Communities.” Scientific Reports 9, no. 1: 5233. 10.1038/s41598-019-41695-z. 30914743 PMC6435756

[acel70095-bib-0070] Vijayakumar, K. A. , and G.‐w. Cho . 2022. “Pan‐Tissue Methylation Aging Clock: Recalibrated and a Method to Analyze and Interpret the Selected Features.” Mechanisms of Ageing and Development 204: 111676. 35489615 10.1016/j.mad.2022.111676

[acel70095-bib-0071] Wlodarczyk, A. , I. R. Holtman , M. Krueger , et al. 2017. “A Novel Microglial Subset Plays a Key Role in Myelinogenesis in Developing Brain.” EMBO Journal 36: 3292–3308. 28963396 10.15252/embj.201696056 PMC5686552

[acel70095-bib-0072] Wolf, E. J. , F. G. Morrison , D. R. Sullivan , et al. 2019. “The Goddess Who Spins the Thread of Life: Klotho, Psychiatric Stress, and Accelerated Aging.” Brain, Behavior, and Immunity 80: 193–203. 30872092 10.1016/j.bbi.2019.03.007 PMC6660403

[acel70095-bib-0073] Ximerakis, M. , S. L. Lipnick , B. T. Innes , et al. 2019. “Single‐Cell Transcriptomic Profiling of the Aging Mouse Brain.” Nature Neuroscience 22, no. 10: 1696–1708. 31551601 10.1038/s41593-019-0491-3

[acel70095-bib-0074] Yi, H. , and N. Stanley . 2021. “Cytoset: Predicting Clinical Outcomes via Set‐Modeling of Cytometry Data.” In: Proceedings of the 12th ACM Conference on Bioinformatics, Computational Biology, and Health Informatics. pp. 1–8.

[acel70095-bib-0075] Yi, H. , and N. Stanley . 2023. “Sclkme: A Landmark‐Based Approach for Generating Multicellular Sample Embeddings From Single‐Cell Data.” bioRxiv. 10.1101/2023.11.13.566846.

[acel70095-bib-0076] Zakar‐Polyák, E. , A. Csordas , R. Pálovics , and C. Kerepesi . 2024. “Profiling the Transcriptomic Age of Single‐Cells in Humans.” Communications Biology 7: 1397. 39462118 10.1038/s42003-024-07094-5 PMC11513945

[acel70095-bib-0077] Zannas, A. S. 2019. “Epigenetics as a Key Link Between Psychosocial Stress and Aging: Concepts, Evidence, Mechanisms.” Dialogues in Clinical Neuroscience 21, no. 4: 389–396. 31949406 10.31887/DCNS.2019.21.4/azannas PMC6952744

[acel70095-bib-0078] Zannas, A. S. , M. Jia , K. Hafner , et al. 2019. “Epigenetic Upregulation of fkbp5 by Aging and Stress Contributes to Nf‐κb–Driven Inflammation and Cardiovascular Risk.” Proceedings of the National Academy of Sciences of the United States of America 116, no. 23: 11370–11379. 31113877 10.1073/pnas.1816847116 PMC6561294

[acel70095-bib-0079] Zhu, H. , J. Chen , K. Liu , et al. 2023. “Human PBMC scRNA‐Seq–Based Aging Clocks Reveal Ribosome to Inflammation Balance as a Single‐Cell Aging Hallmark and Super Longevity.” Science Advances 9: eabq7599. 37379396 10.1126/sciadv.abq7599 PMC10306289

