# Supplementary material for: Microglia Single‐Cell RNA‐Seq Enables Robust and Applicable Markers of Biological Aging
Source: Aging Cell. 2025 May 15;24(8):e70095. doi: 10.1111/acel.70095 (PMC12341818; doi:10.1111/acel.70095)
Supplement: Supplementary file 1 — Data S1. [file ACEL-24-e70095-s001.zip › Supplement_Stanley_Dhawka_Zannas_AgingCell.pdf]

Supplementary Material

March 27, 2025

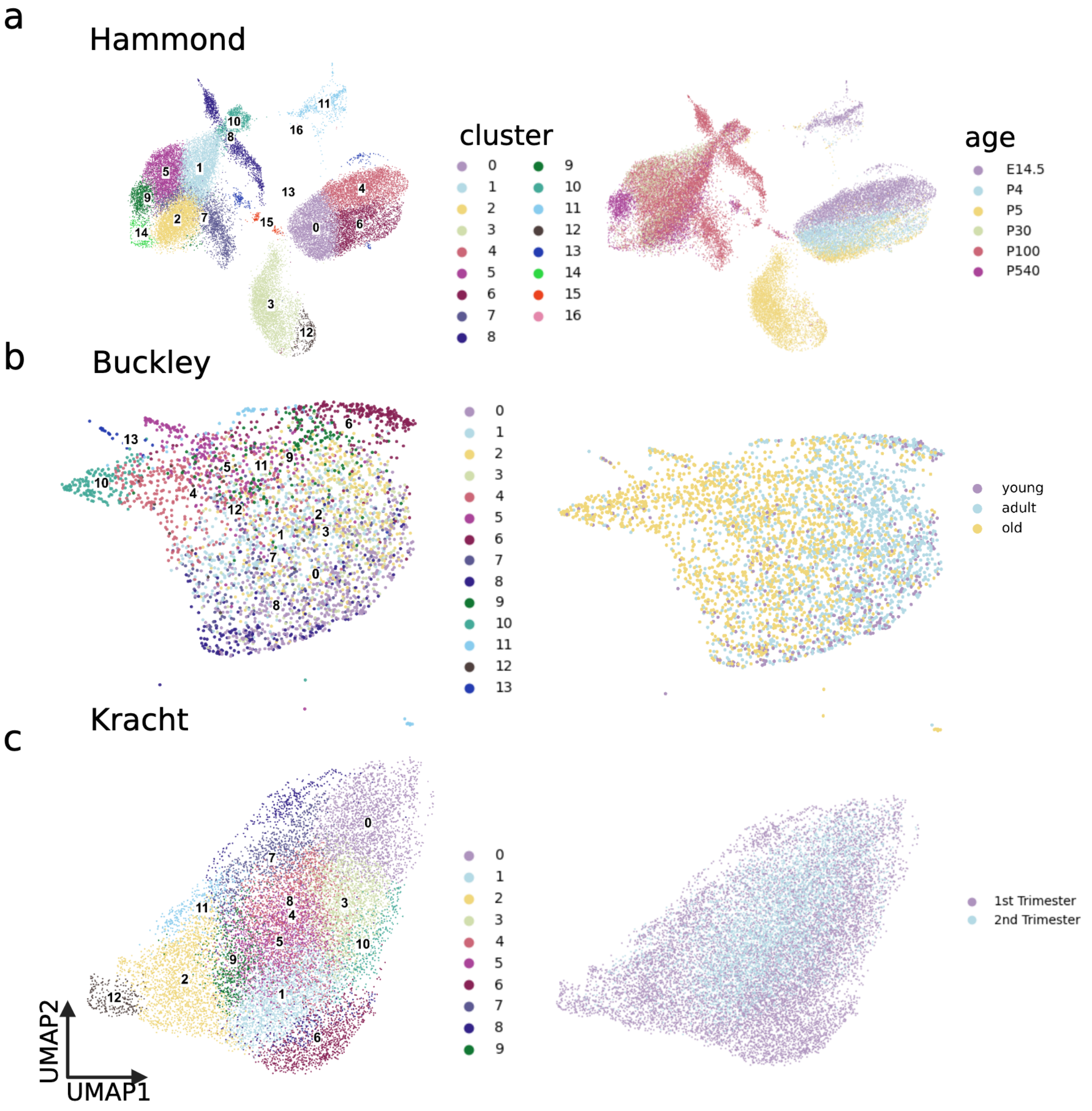

Figure 1: **Clustering and two-dimensional visualization of cells across datasets reveal microglia subtypes with characteristic genetic programs**. Cells were projected in two dimensions with UMAP and colored according to Leiden cluster (**left**) and age group (**right**) in the Hammond **a**, Buckley **b**, and Kracht **c** datasets.

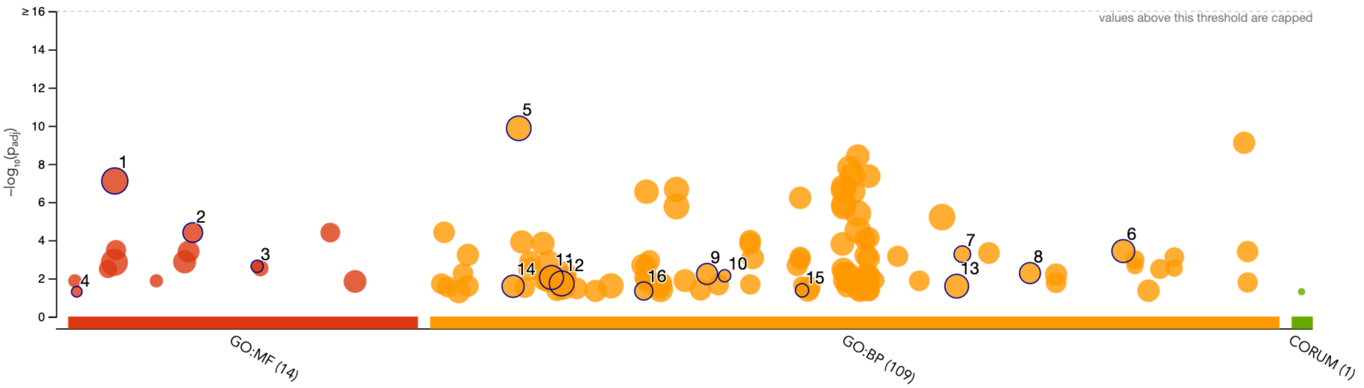

ID	Source	Term ID	Term Name	p_{adj} (query_1)
1	GO:MF	GO:0005515	protein binding	7.829×10^{-8}
2	GO:MF	GO:0030695	GTPase regulator activity	3.968×10^{-5}
3	GO:MF	GO:0045028	G protein-coupled purinergic nucleotide recept...	2.330×10^{-3}
4	GO:MF	GO:0001875	lipopolysaccharide immune receptor activity	4.971×10^{-2}
5	GO:BP	GO:0007275	multicellular organism development	1.355×10^{-10}
6	GO:BP	GO:1901700	response to oxygen-containing compound	3.695×10^{-4}
7	GO:BP	GO:0071277	cellular response to calcium ion	5.385×10^{-4}
8	GO:BP	GO:0097435	supramolecular fiber organization	5.283×10^{-3}
9	GO:BP	GO:0034330	cell junction organization	5.848×10^{-3}
10	GO:BP	GO:0035589	G protein-coupled purinergic nucleotide recept...	7.294×10^{-3}
11	GO:BP	GO:0010033	response to organic substance	9.026×10^{-3}
12	GO:BP	GO:0010468	regulation of gene expression	1.834×10^{-2}
13	GO:BP	GO:0070887	cellular response to chemical stimulus	2.559×10^{-2}
14	GO:BP	GO:0007010	cytoskeleton organization	2.588×10^{-2}
15	GO:BP	GO:0045655	regulation of monocyte differentiation	4.214×10^{-2}
16	GO:BP	GO:0022408	negative regulation of cell-cell adhesion	4.588×10^{-2}

Figure 2: **Gene ontology results for genes in module 1 of the Hammond dataset under DELVE.** Top enriched GO-terms among genes in dynamic module 1 (increasing with age) obtained with DELVE in the Hammond dataset.

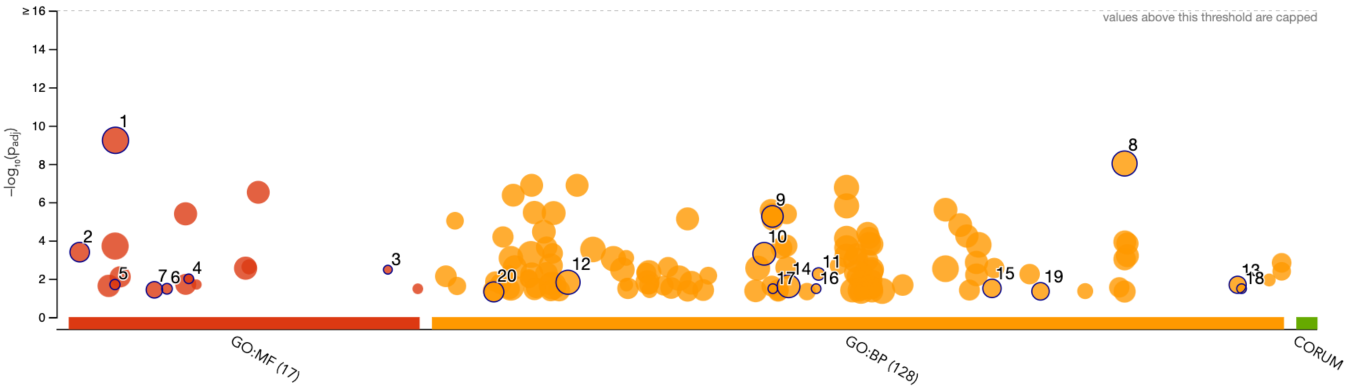

ID	Source	Term ID	Term Name	p_{adj} (query_1)
1	GO:MF	GO:0005515	protein binding	5.872×10^{-10}
2	GO:MF	GO:0003729	mRNA binding	4.058×10^{-4}
3	GO:MF	GO:0106130	purine phosphoribosyltransferase activity	3.345×10^{-3}
4	GO:MF	GO:0030060	L-malate dehydrogenase activity	1.002×10^{-2}
5	GO:MF	GO:0005471	ATP:ADP antiporter activity	2.000×10^{-2}
6	GO:MF	GO:0017077	oxidative phosphorylation uncoupler activity	3.328×10^{-2}
7	GO:MF	GO:0016209	antioxidant activity	3.766×10^{-2}
8	GO:BP	GO:1901564	organonitrogen compound metabolic process	9.509×10^{-9}
9	GO:BP	GO:0043066	negative regulation of apoptotic process	5.510×10^{-6}
10	GO:BP	GO:0042592	homeostatic process	4.956×10^{-4}
11	GO:BP	GO:0046166	glyceraldehyde-3-phosphate biosynthetic pro...	5.629×10^{-3}
12	GO:BP	GO:0010646	regulation of cell communication	1.519×10^{-2}
13	GO:BP	GO:1905954	positive regulation of lipid localization	2.061×10^{-2}
14	GO:BP	GO:0044419	biological process involved in interspecies inte...	2.455×10^{-2}
15	GO:BP	GO:0072330	monocarboxylic acid biosynthetic process	3.175×10^{-2}
16	GO:BP	GO:0046083	adenine metabolic process	3.278×10^{-2}
17	GO:BP	GO:0043096	purine nucleobase salvage	3.278×10^{-2}
18	GO:BP	GO:1990428	miRNA transport	3.278×10^{-2}
19	GO:BP	GO:0098754	detoxification	4.522×10^{-2}
20	GO:BP	GO:0006091	generation of precursor metabolites and energy	4.838×10^{-2}

Figure 3: **Gene ontology results for genes in module 2 of the Hammond dataset under DELVE.** Top enriched GO-terms among genes in dynamic module 2 (decreasing with age) obtained with DELVE in the Hammond dataset.

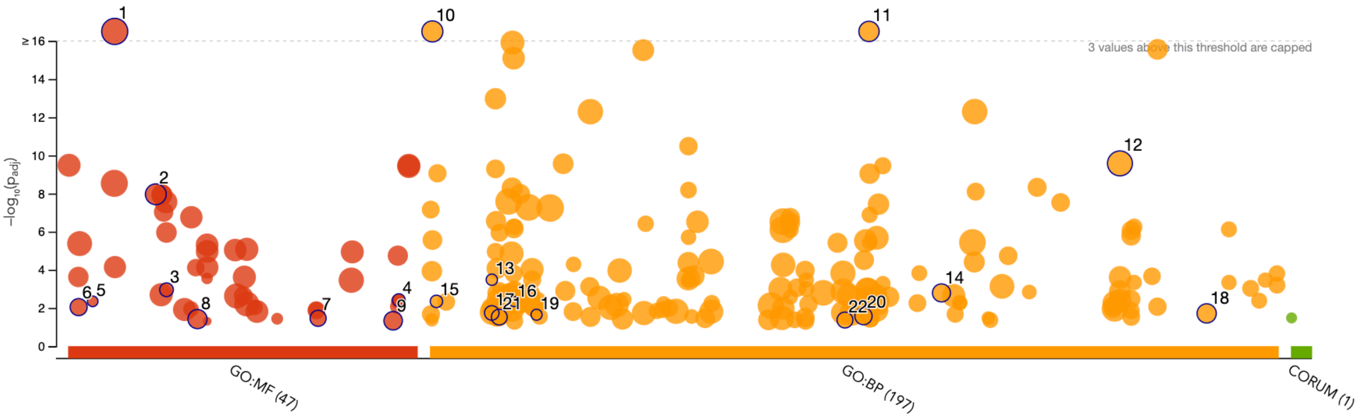

ID	Source	Term ID	Term Name	p_{adj} (query_1)
1	GO:MF	GO:0005515	protein binding	4.243×10^{-18}
2	GO:MF	GO:0016462	pyrophosphatase activity	1.092×10^{-8}
3	GO:MF	GO:0017116	single-stranded DNA helicase activity	1.111×10^{-3}
4	GO:MF	GO:0140713	histone chaperone activity	3.871×10^{-3}
5	GO:MF	GO:0004448	isocitrate dehydrogenase [NAD(P)+] activity	4.490×10^{-3}
6	GO:MF	GO:0003697	single-stranded DNA binding	9.014×10^{-3}
7	GO:MF	GO:0051536	iron-sulfur cluster binding	3.478×10^{-2}
8	GO:MF	GO:0031625	ubiquitin protein ligase binding	3.829×10^{-2}
9	GO:MF	GO:0140097	catalytic activity, acting on DNA	4.675×10^{-2}
10	GO:BP	GO:0000278	mitotic cell cycle	5.016×10^{-18}
11	GO:BP	GO:0051276	chromosome organization	2.416×10^{-17}
12	GO:BP	GO:1901564	organonitrogen compound metabolic process	2.626×10^{-10}
13	GO:BP	GO:0006102	isocitrate metabolic process	3.295×10^{-4}
14	GO:BP	GO:0065004	protein-DNA complex assembly	1.621×10^{-3}
15	GO:BP	GO:0000727	double-strand break repair via break-induced r...	4.507×10^{-3}
16	GO:BP	GO:0007057	spindle assembly involved in female meiosis I	4.572×10^{-3}
17	GO:BP	GO:0006099	tricarboxylic acid cycle	1.819×10^{-2}
18	GO:BP	GO:1904951	positive regulation of establishment of protein l...	1.924×10^{-2}
19	GO:BP	GO:0009211	pyrimidine deoxyribonucleoside triphosphate ...	2.257×10^{-2}
20	GO:BP	GO:0051028	mRNA transport	2.674×10^{-2}
21	GO:BP	GO:0006446	regulation of translational initiation	3.003×10^{-2}
22	GO:BP	GO:0048599	oocyte development	4.288×10^{-2}

Figure 4: **Gene ontology results for genes in module 3 of the Hammond dataset under DELVE.** Top enriched GO-terms among genes in dynamic module 3 (marginally decreasing with age) obtained with DELVE in the Hammond dataset.

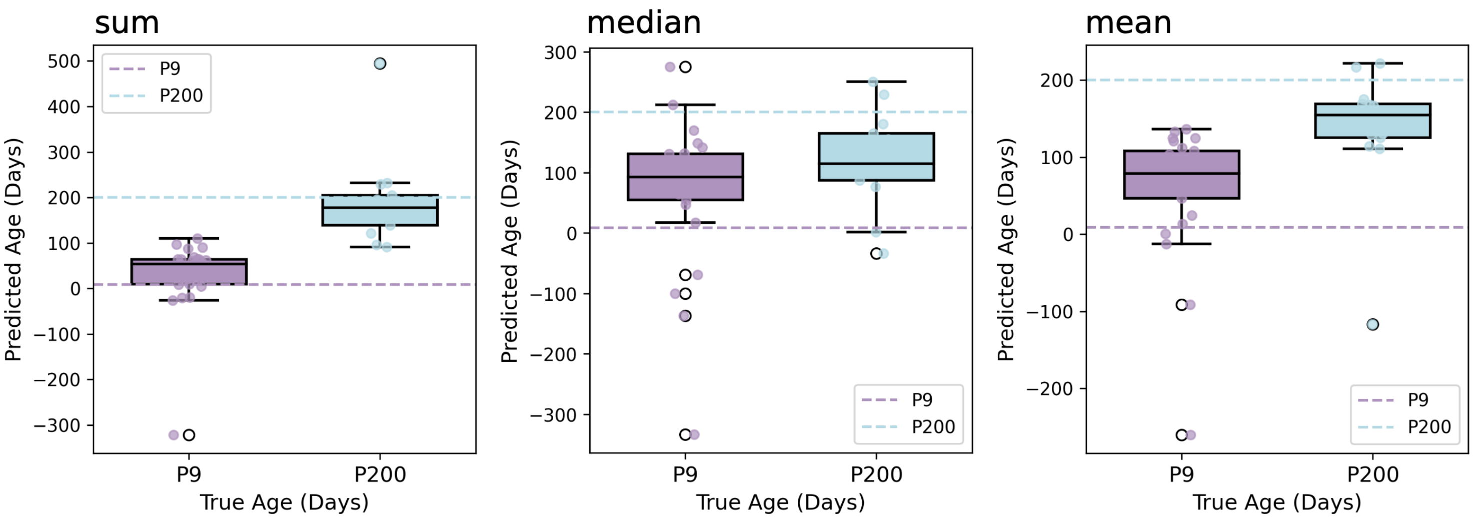

Figure 5: **Possible pooling methods for generating simplified pseudobulk.** Various pooling approaches, including sum (**left**), median (**center**), and mean (**right**) were explored for converting single-cell profiles into a simplified pseudobulk data structure. Briefly, these approaches indicate how the expression of genes are aggregated across cells in samples profiled with single-cell to create a sample $\times$ gene data structure that can be used to train a model, which can be applied to a bulk RNA-seq dataset.

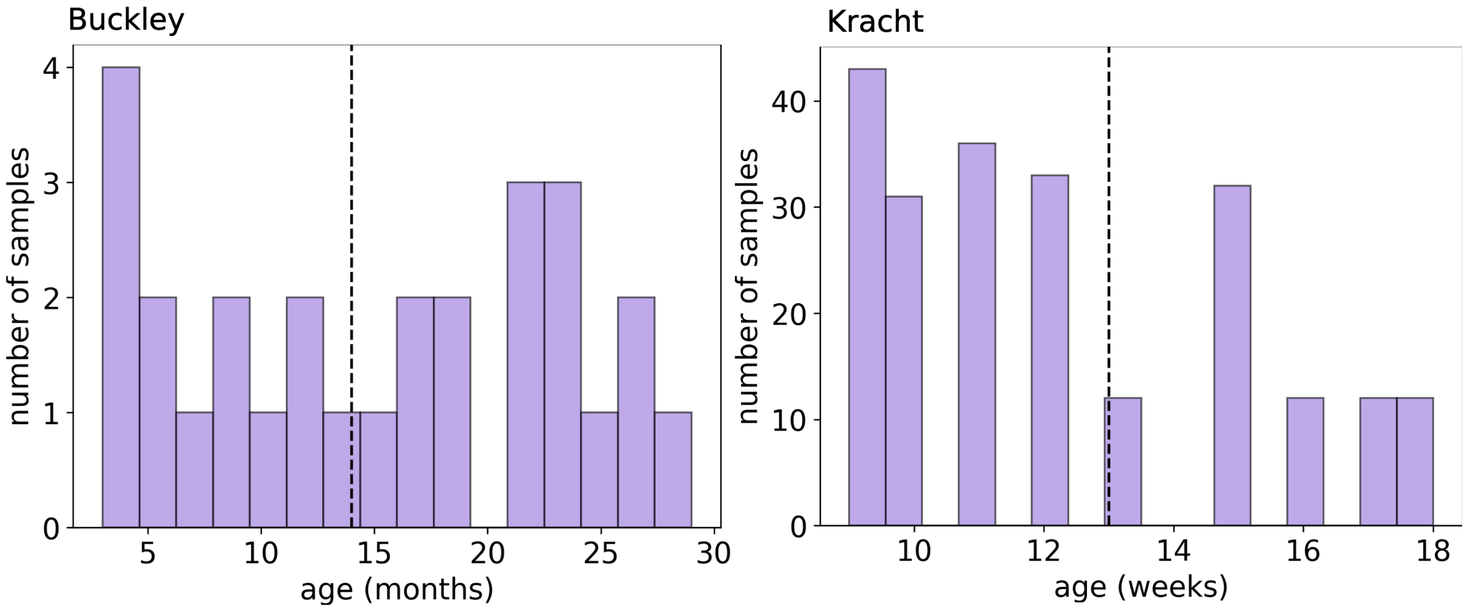

Figure 6: **Distribution of age in the Buckley and Kracht datasets.** To formulate age classification problems, ages in the Buckley and Kracht datasets were binned into two discrete age categories. Histograms show the distribution of edges across samples and the dotted vertical line shows the age threshold used to bin samples by age. Note that samples were split by trimester (first vs second) in the Kracht dataset.

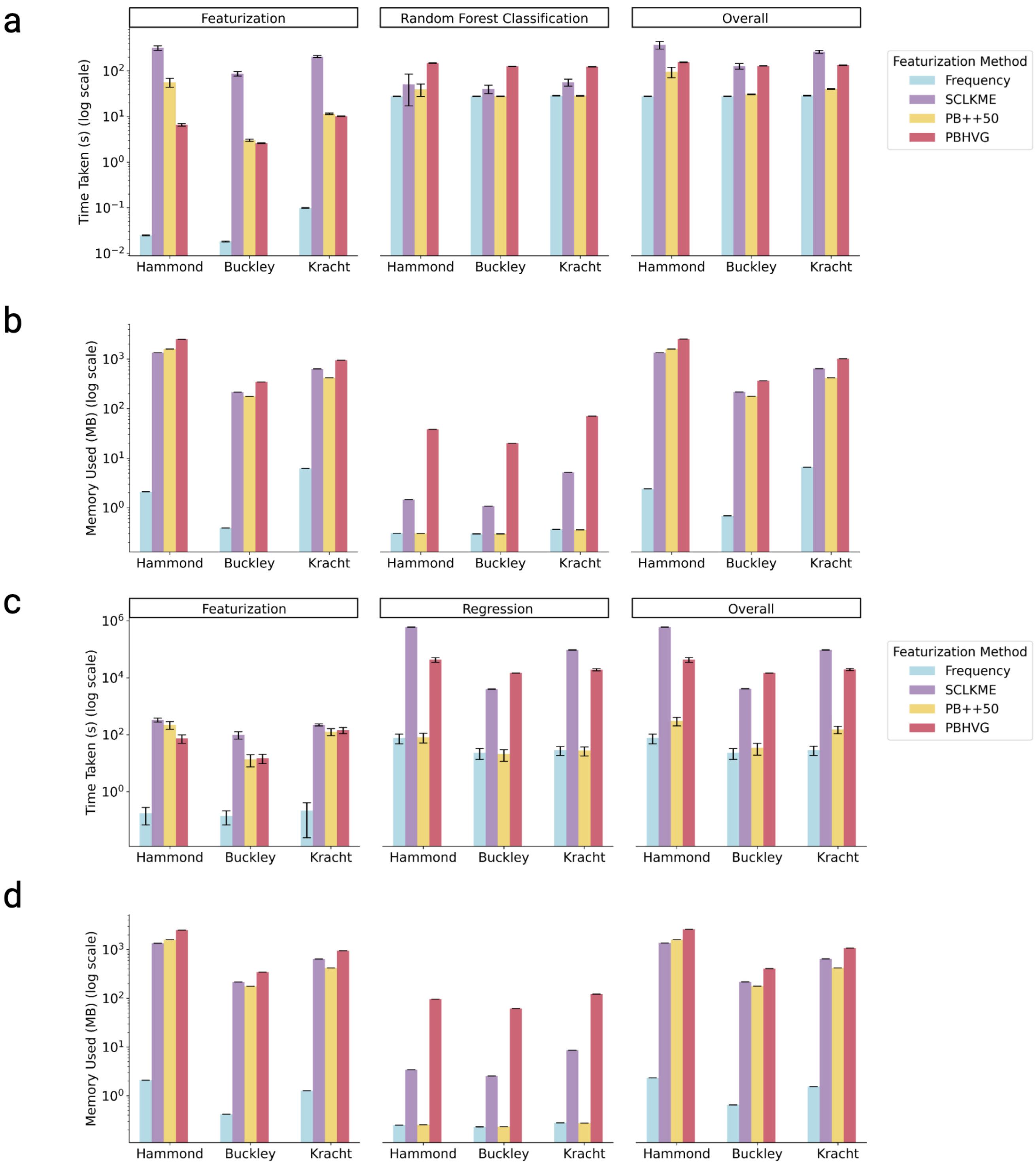

Figure 7: **Run-time and memory requirements for featurization and aging clocks.** Run-time (**a,c**) and memory (**b,d**) required for each featurization approach were evaluated across datasets for featurization only (**left panels**), classification with Random Forest (**middle a,b**) or regression with Lasso (**middle c,d**) to build clocks, and the overall process of featurization and classification (**panels a-b**) or regression (**panels c-d**). Results were obtained by repeating the featurization and model training over 30 trials and reporting the mean. Error bars show standard deviation around the mean.

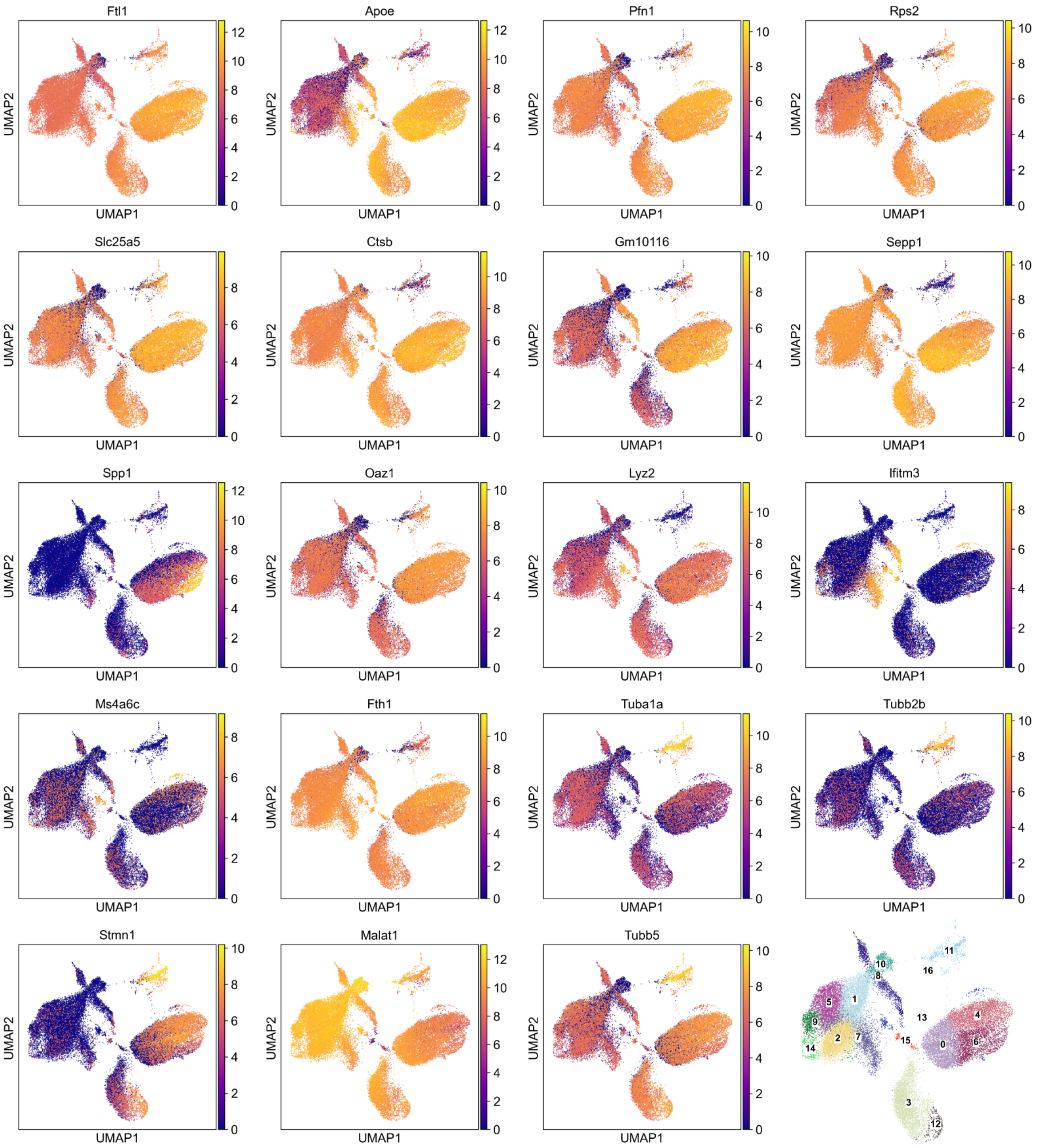

Figure 8: **Key differentially expressed genes across clusters in the Hammond dataset.** Cells from all samples in the Hammond dataset were projected in two dimensions with UMAP and colored by the expression of key cluster-specific differentially expressed genes.

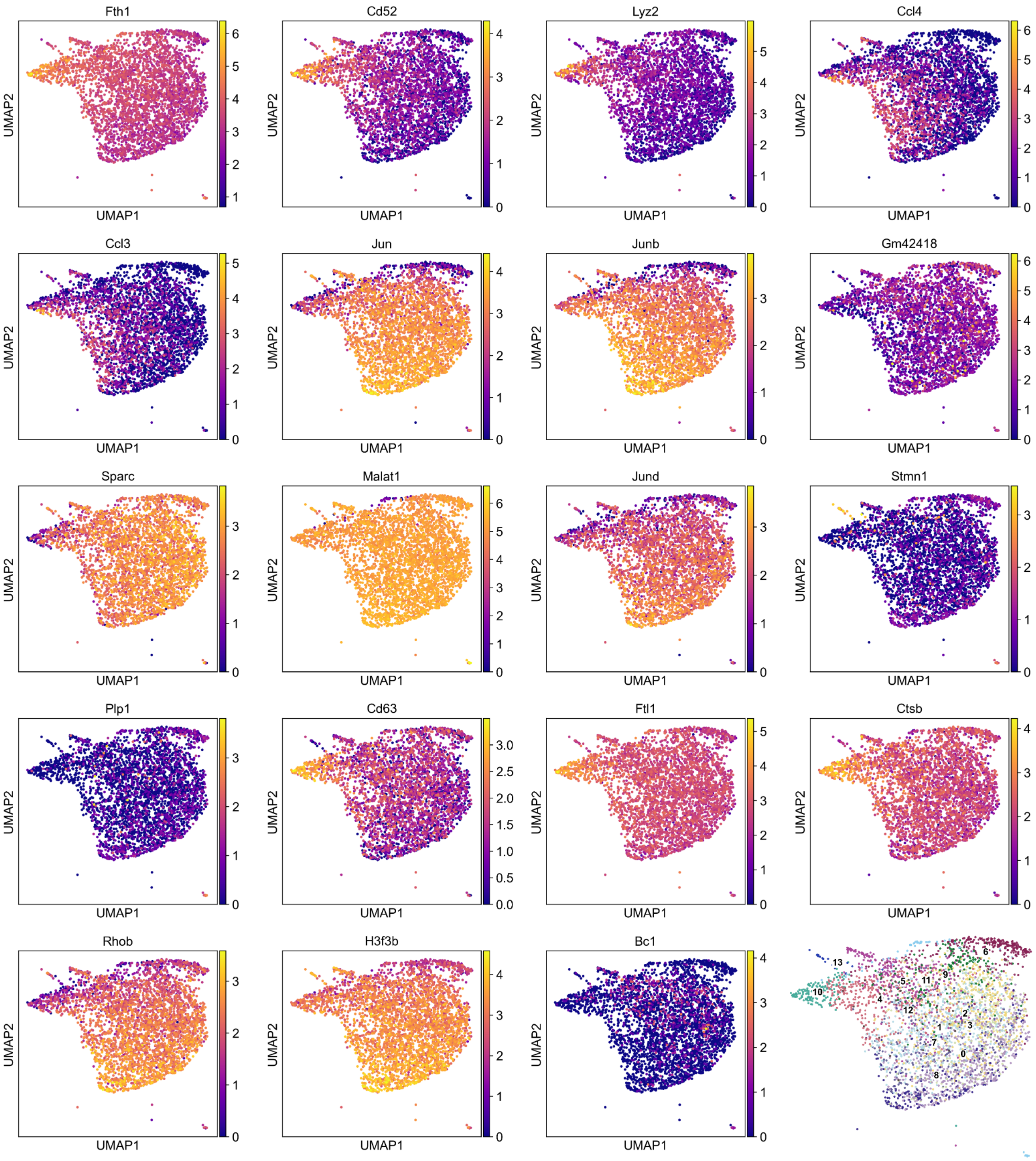

Figure 9: **Key differentially expressed genes across clusters in the Buckley dataset.** Cells from all samples in the Buckley dataset were projected in two dimensions with UMAP and colored by the expression of key cluster-specific differentially expressed genes.

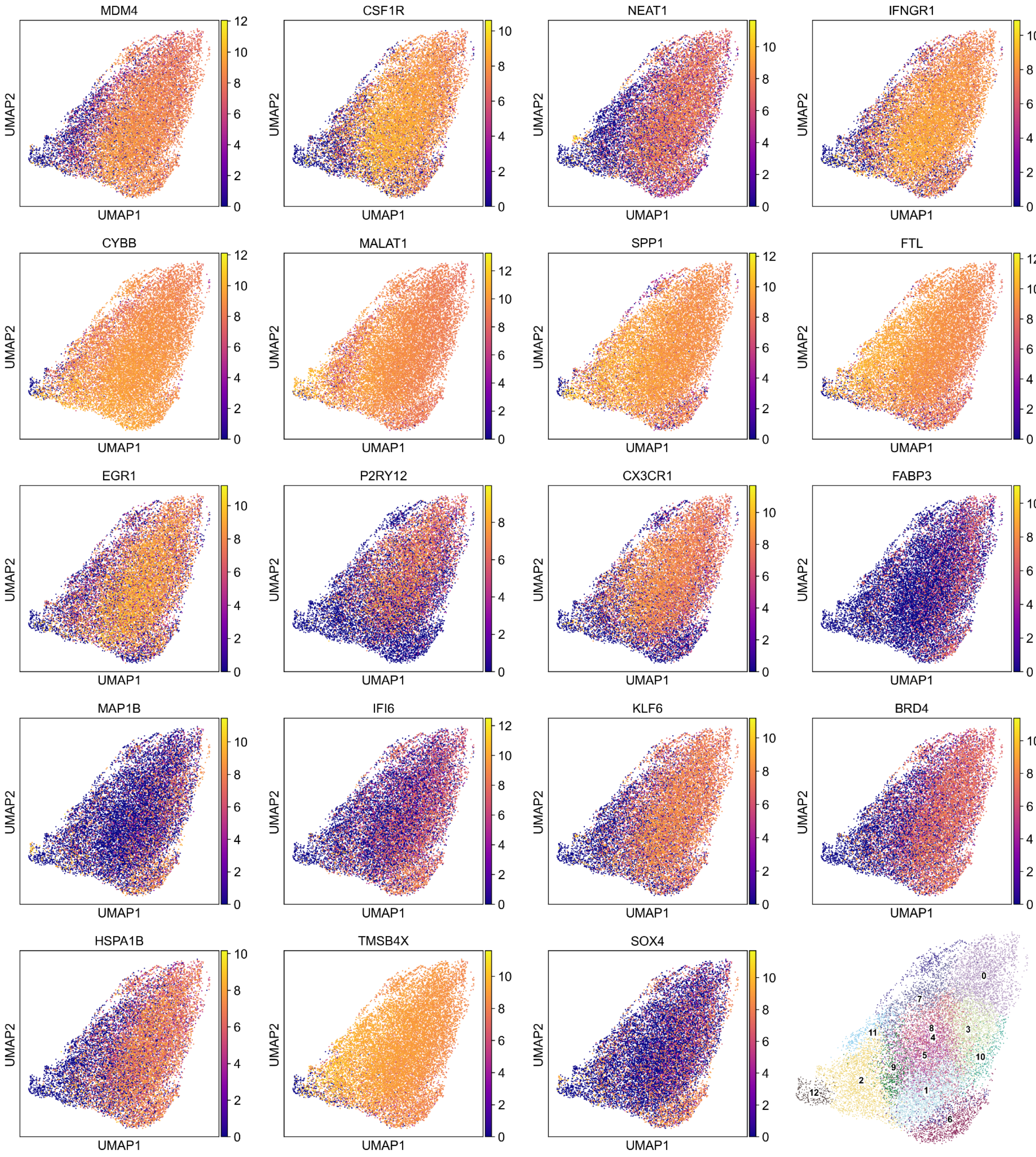

Figure 10: **Key differentially expressed genes across clusters in the Kracht dataset.** Cells from all samples in the Kracht dataset were projected in two dimensions with UMAP and colored by the expression of key cluster-specific differentially expressed genes.

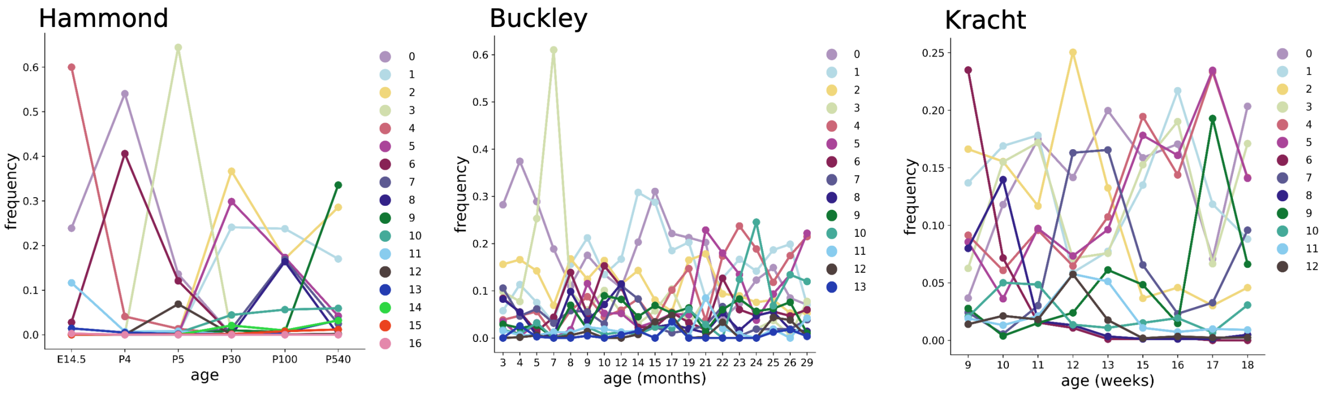

Figure 11: **Frequencies of each cluster plotted as a function of age.** Mean frequencies of each identified cluster across samples in the Hammond, Buckley, and Kracht datasets were plotted as a function of age. Line colors indicate cluster number in their respective datasets. Patterns of increase or decrease of particular clusters can be cross-checked with trajectories highlighted for their relevance for age prediction in Fig. 2. See supplementary tables 1,2, and 3 for an annotation of the clusters in each respective dataset.

cluster	microglia subtype	activity with aging/development
0	Advanced DAM and Inflam.DAM	decrease
1	Homeos1 and Homeos3	increase
2	Homeos1	increase
3	Homeos1 and Inflam.DAM	Peak at P5 then decrease
4	DIMs and Proliferating	decrease
5	Homeos1	Peak at P30, then decrease
6	Neonatal_CD11c and ATM	decrease
7	IRM	Peak at P100, then decrease
8	Homeos1 and Homeos2	Peak at P100, then decrease
9	Homeos3 and DIMs	increase
10	Homeos1 and Homeos2	increase
11	Proliferating and Ribo.DAM2	decrease
12	Proliferating	Peak at P5 then decrease
13	Border Associated Macrophage (MAC)	increase
14	Homeos1 and Homeos2	increase
15	IRM	increase
16	Proliferating	constant, low frequency

Table 1: Annotations of microglia subtypes (clusters) identified in the Hammond dataset and their pattern with aging.

cluster	microglia subtype	activity with aging
0	Homeos1 and Homeos2 and Homeos3	cyclical
1	Homeos3	decrease after 19 months
2	Homeos1 and Homeos3	constant
3	Homeos1 and Homeos2 and Homeos3	decrease after 7 months
4	DIMs and IRM	increase after 21 months
5	IRM	cyclical, but increasing after 24 months
6	Homeos1 and Homeos2	constant
7	Homeos2	decrease
8	Homeos2	decrease
9	Inflam.DAM and ARM	constant
10	ATM and IRM	increase
11	Macrophage (MAC)	constant low frequency
12	Homeos3 and Advanced DAM	constant low frequency
13	Proliferating	constant low frequency

Table 2: Annotations of microglia subtypes (clusters) identified in the Buckley dataset and their pattern with aging.

cluster	microglia subtype	activity with development
0	Homeos2 and Ribo.DAM1	cyclical
1	Homeos1 and Homeos3 and Inflam.DAM	cyclical
2	Proliferating and ATM	decrease
3	DIMs and Transitional_IFN-DAM	cyclical
4	Homeos1 and Homeos2 and Homeos3	increase
5	Homeos1	increase
6	Proliferating and DIMs	decrease
7	Ribo.DAM2 and ARM	cyclical
8	MAC and MHCII-high	decrease
9	Homeos1 and Homeos2 and Homeos3	increase
10	DIMs and Embryonic	decrease
11	Ribo.DAM2 and Proliferating	decrease
12	Proliferating and Homeos3	decrease

Table 3: Annotations of microglia subtypes (clusters) identified in the Kracht dataset and their pattern with development.

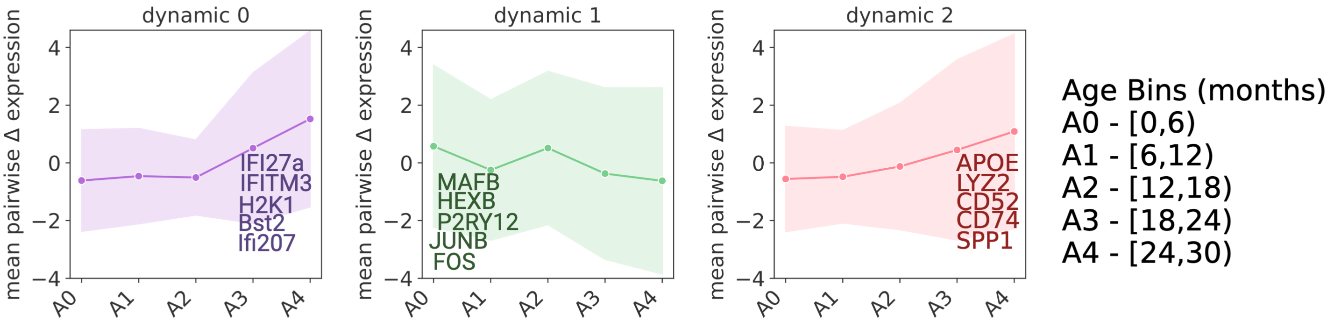

Figure 12: **DELVE applied to the Buckley dataset reveals 3 dynamic gene modules.** Three modules with characteristic dynamic expression patterns were identified with DELVE in the Buckley dataset by creating four discrete age bins. Five significant genes in each module are shown. Dynamic module 0 shows several inflammatory genes with expression that increases with age.

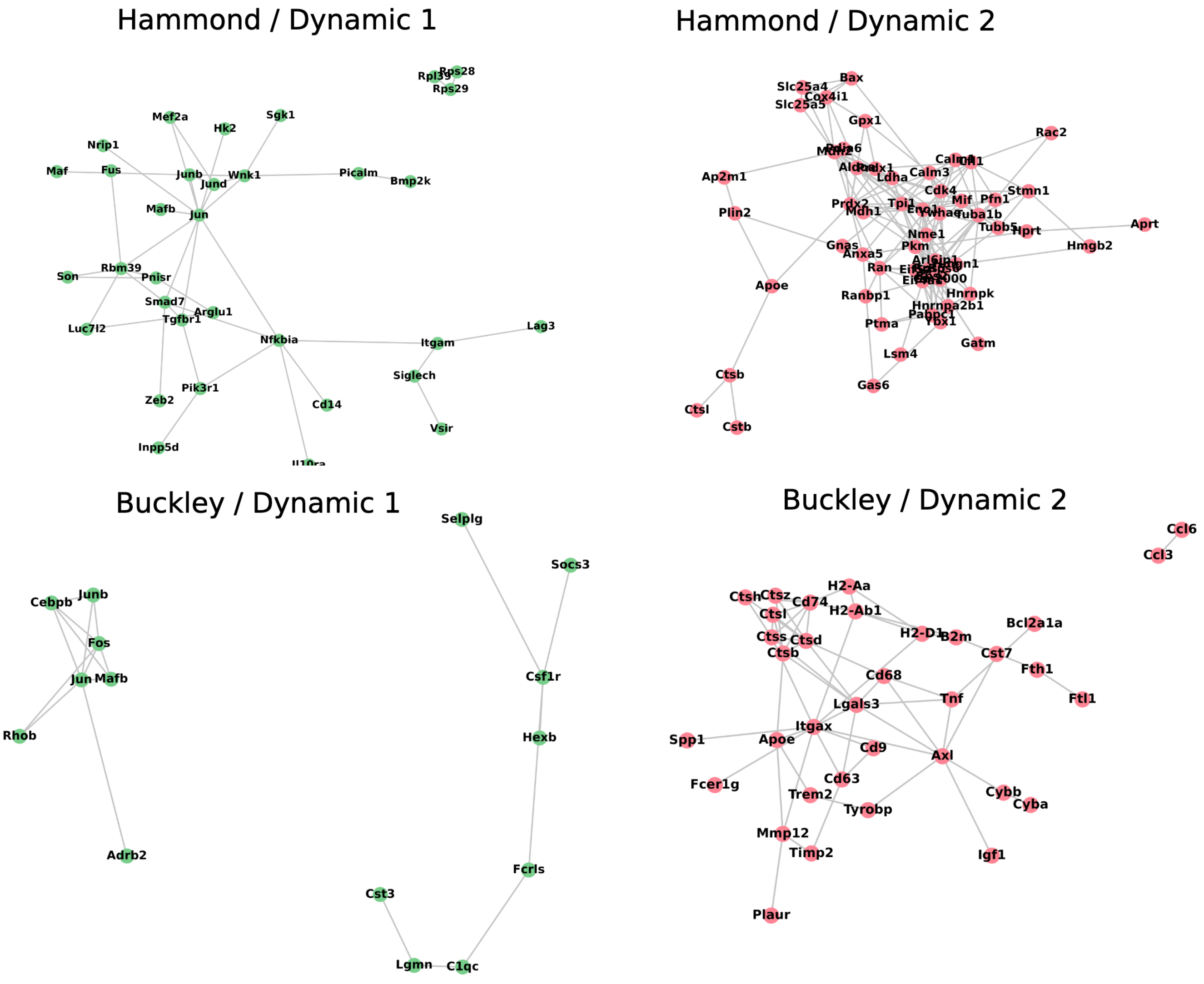

Figure 13: **Gene regulatory networks emerge from DELVE modules.** Gene regulatory networks were constructed from the STRING database for DELVE modules 1 and 2 in the Hammond (see DELVE module trends in Fig. 6) and Buckley (see DELVE module trends in Supplementary Fig. 12) datasets. See tables entitled DELVE_modules_Buckley.csv and DELVE_modules_Hammond.csv in Additional Information for gene-to-module assignments.

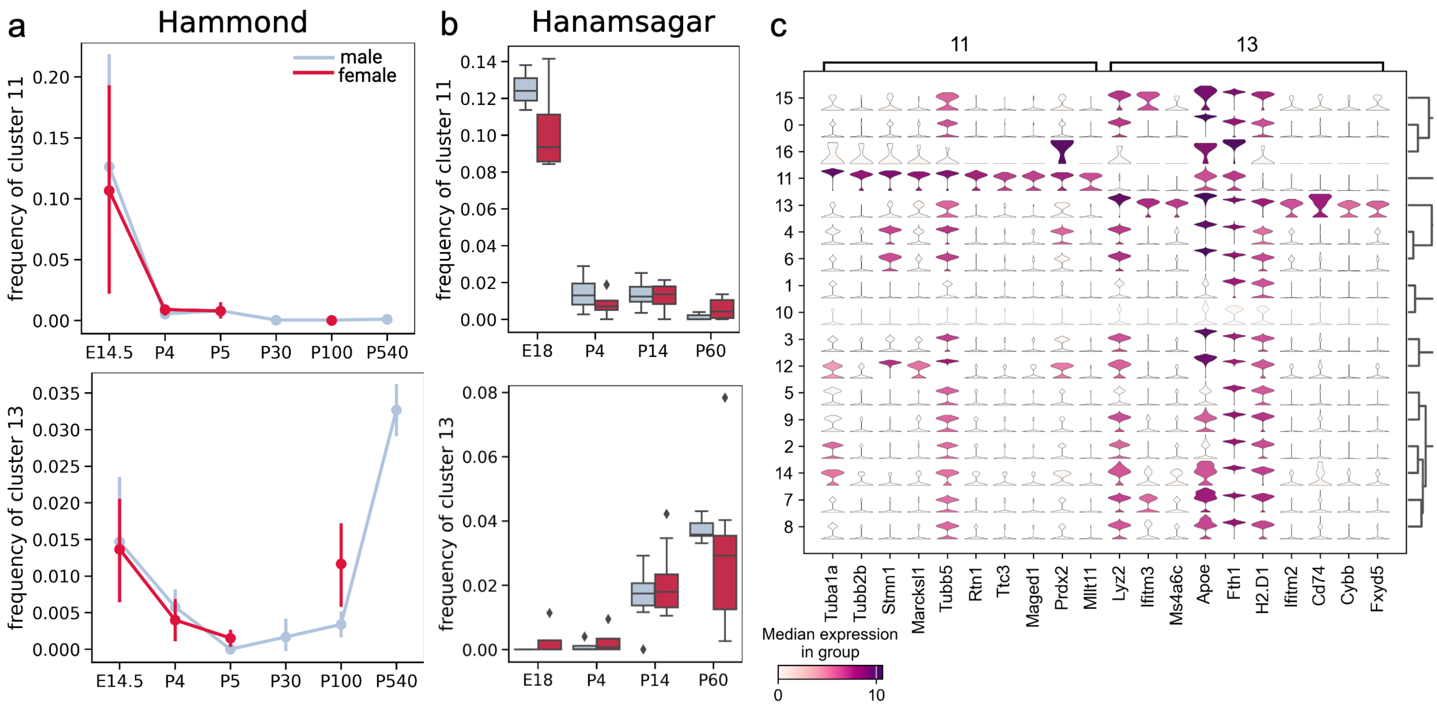

Figure 14: **Evaluating sex-differences in Hammond subtypes.** Cibersort was used to deconvolve bulk RNA sequencing signatures in the Hanamsagar dataset based on Hammond subtypes. Clusters 11 (proliferating microglia) and 13 (macrophages) were prioritized because their frequency patterns were the same across lifespan between the Hammond and Hanamsagar datasets. **a** Plots show the mean trajectory of frequencies of clusters 11 and 13 throughout lifespan in the Hammond dataset in males (blue) and females (pink). Points show mean and error bars show standard error. **b** Boxplots show the distribution of deconvolved frequencies in males (blue) and females (pink) in the Hanamsagar dataset. There were no statistically significant frequency differences between males in females ($p = 0.2209$ for sex, $p = 0.0649$ for age/sex interactions in cluster 11 ; $p = 0.889$ for sex, $p = 0.587$ for age/sex interactions in cluster 13). **c** Violin plots visualize the top 10 differentially expressed genes in clusters 11 and 13 and their expression distributions across all other clusters.

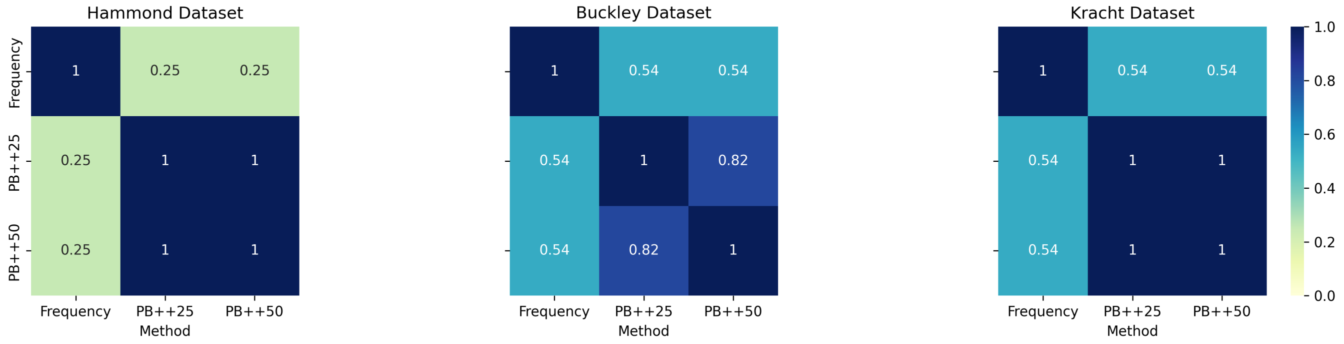

Figure 15: **Similarity in top age-predictive features between featurization approaches.** Heatmaps show the Jaccard similarity between the set of top 10 clusters identified by training a random forest classifier for age, under each frequency-based featurization approach. Jaccard similarity reflects the fraction of clusters ranked as the top 10 most predictive according to Gini score between a given pair of methods.

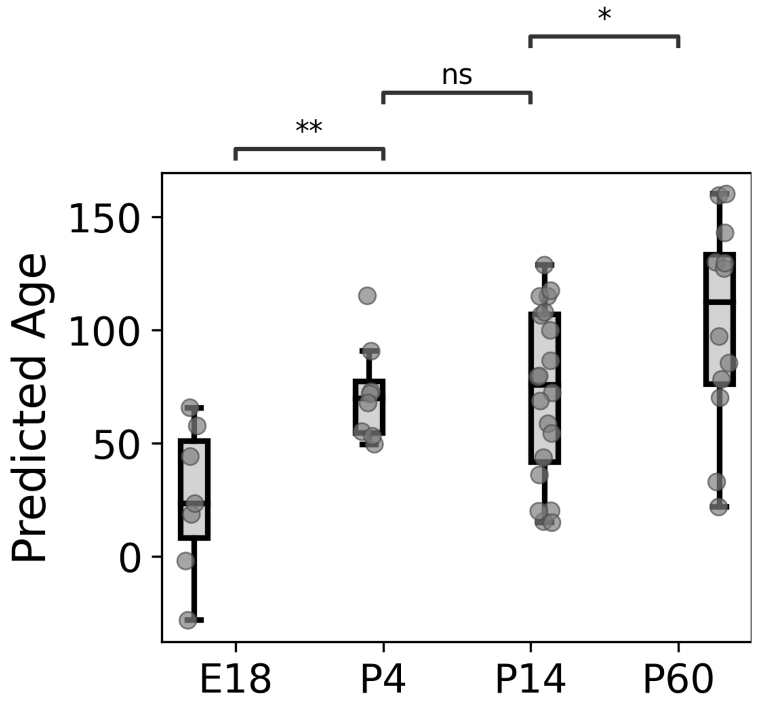

Figure 16: **The bulk-to-single-cell extrapolation approach in the Hanamsagar dataset.** A Hammond-trained pseudobulk-based clock was used to predict age in the Hanamsagar dataset. There were statistically-significant differences in predicted ages between pairs of adjacent age groups, including, E18 and P4 ($p = 0.0068$), and P14 and P60 ($p = 0.047$).

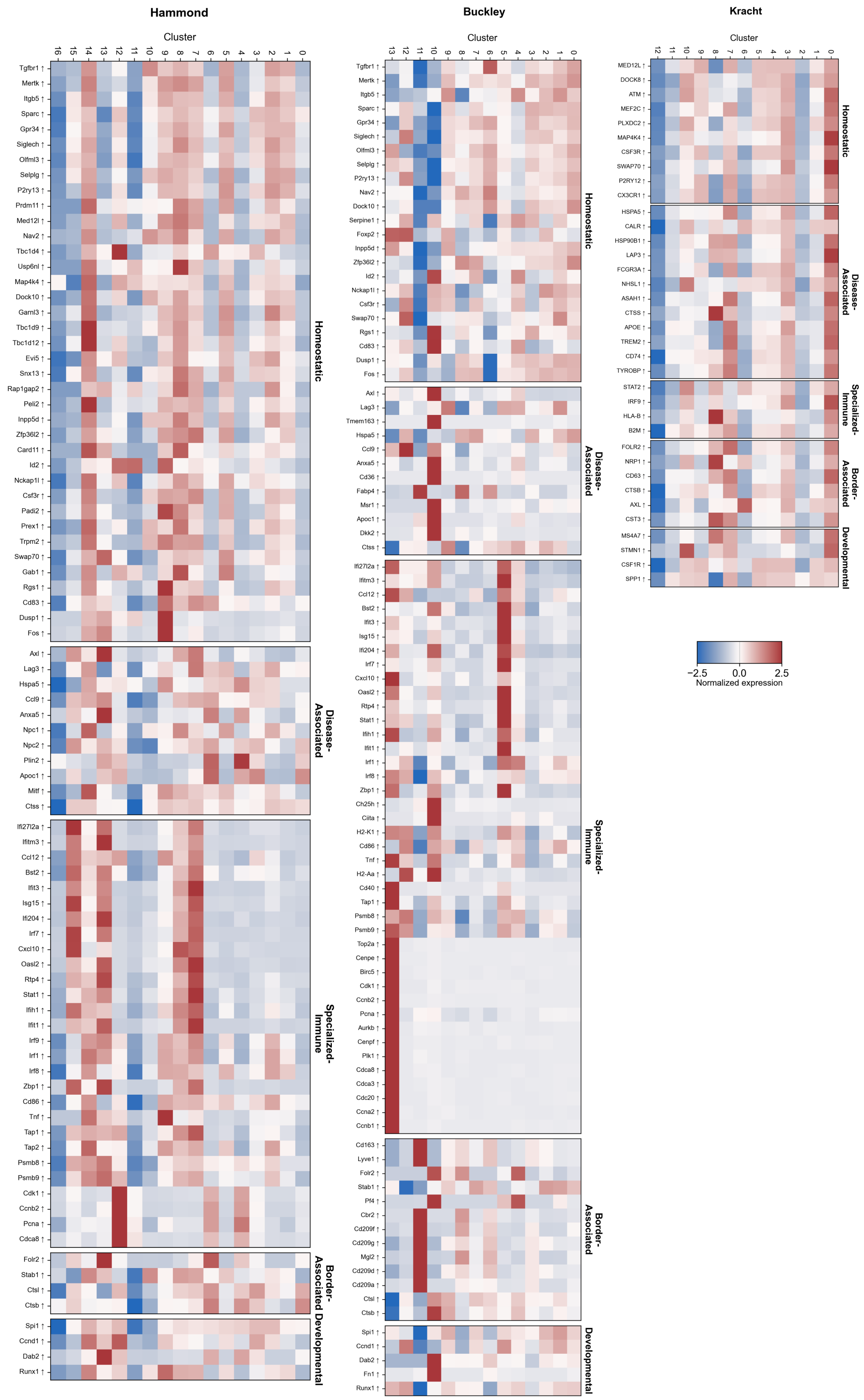

Figure 17: **Gene expression patterns of microglia sub-types across datasets.** Heatmaps visualize the expression of the key marker genes for each microglia subtype across datasets. Microlia subtypes are described in the Additional Information file entitled `Microglia_Subtype_Stanley_Dhawka_Zannas.csv`.
